# Extracellular vesicles released by glioblastoma cancer cells drive tumor invasiveness via Connexin-43 gap junctions

**DOI:** 10.1093/neuonc/noaf013

**Published:** 2025-01-30

**Authors:** Matteo Tamborini, Valentino Ribecco, Elisabetta Stanzani, Arianna Sironi, Monica Tambalo, Davide Franzone, Elena Florio, Edoardo Fraviga, Chiara Saulle, Maria C Gagliani, Marco Pizzocri, Milena Mattioli, Katia Cortese, Jean X Jiang, Giuseppe Martano, Letterio S Politi, Marco Riva, Federico Pessina, Davide Pozzi, Simona Lodato, Lorena Passoni, Michela Matteoli

**Affiliations:** CNR Institute of Neuroscience c/o IRCCS Humanitas Research Hospital, Rozzano, Milano, 20089, Italy; IRCCS Humanitas Research Hospital, Rozzano, Milano, 20089, Italy; IRCCS Humanitas Research Hospital, Rozzano, Milano, 20089, Italy; Department of Biomedical Sciences, Humanitas University, Pieve Emanuele, Milano, 20072, Italy; CNR Institute of Neuroscience c/o IRCCS Humanitas Research Hospital, Rozzano, Milano, 20089, Italy; IRCCS Humanitas Research Hospital, Rozzano, Milano, 20089, Italy; Department of Experimental Medicine (DIMES), Cellular Electron Microscopy Laboratory, Università di Genova, Genova, 16132, Italy; Department of Biomedical Sciences, Humanitas University, Pieve Emanuele, Milano, 20072, Italy; IRCCS Humanitas Research Hospital, Rozzano, Milano, 20089, Italy; Department of Biomedical Sciences, Humanitas University, Pieve Emanuele, Milano, 20072, Italy; IRCCS Humanitas Research Hospital, Rozzano, Milano, 20089, Italy; Department of Biomedical Sciences, Humanitas University, Pieve Emanuele, Milano, 20072, Italy; IRCCS Humanitas Research Hospital, Rozzano, Milano, 20089, Italy; Department of Biomedical Sciences, Humanitas University, Pieve Emanuele, Milano, 20072, Italy; IRCCS Humanitas Research Hospital, Rozzano, Milano, 20089, Italy; Department of Biomedical Sciences, Humanitas University, Pieve Emanuele, Milano, 20072, Italy; IRCCS Humanitas Research Hospital, Rozzano, Milano, 20089, Italy; Department of Biomedical Sciences, Humanitas University, Pieve Emanuele, Milano, 20072, Italy; IRCCS Humanitas Research Hospital, Rozzano, Milano, 20089, Italy; Department of Biochemistry and Structural Biology, University of Texas Health Science Center at San Antonio, San Antonio, Texas 78229, USA; Department of Experimental Medicine (DIMES), Cellular Electron Microscopy Laboratory, Università di Genova, Genova, 16132, Italy; IRCCS Humanitas Research Hospital, Rozzano, Milano, 20089, Italy; Department of Biomedical Sciences, Humanitas University, Pieve Emanuele, Milano, 20072, Italy; IRCCS Humanitas Research Hospital, Rozzano, Milano, 20089, Italy; Department of Experimental Medicine (DIMES), Cellular Electron Microscopy Laboratory, Università di Genova, Genova, 16132, Italy; Department of Biochemistry and Structural Biology, University of Texas Health Science Center at San Antonio, San Antonio, Texas 78229, USA; IRCCS Humanitas Research Hospital, Rozzano, Milano, 20089, Italy; Department of Biomedical Sciences, Humanitas University, Pieve Emanuele, Milano, 20072, Italy; IRCCS Humanitas Research Hospital, Rozzano, Milano, 20089, Italy; Department of Biomedical Sciences, Humanitas University, Pieve Emanuele, Milano, 20072, Italy; IRCCS Humanitas Research Hospital, Rozzano, Milano, 20089, Italy; Department of Biomedical Sciences, Humanitas University, Pieve Emanuele, Milano, 20072, Italy; IRCCS Humanitas Research Hospital, Rozzano, Milano, 20089, Italy; Department of Biomedical Sciences, Humanitas University, Pieve Emanuele, Milano, 20072, Italy; IRCCS Humanitas Research Hospital, Rozzano, Milano, 20089, Italy; Department of Biomedical Sciences, Humanitas University, Pieve Emanuele, Milano, 20072, Italy; IRCCS Humanitas Research Hospital, Rozzano, Milano, 20089, Italy; IRCCS Humanitas Research Hospital, Rozzano, Milano, 20089, Italy; Department of Biomedical Sciences, Humanitas University, Pieve Emanuele, Milano, 20072, Italy; IRCCS Humanitas Research Hospital, Rozzano, Milano, 20089, Italy

**Keywords:** assembloids, calcium signaling, Connexin-43, extracellular vesicles, focal adhesion complex, glioblastoma, invasion, migration, stem cells, surgical aspirate

## Abstract

**Background:**

Although invasiveness is one of the major determinants of the poor glioblastoma (GBM) outcome, the mechanisms of GBM invasion are only partially understood. Among the intrinsic and environmental processes promoting cell-to-cell interaction processes, eventually driving GBM invasion, we focused on the pro-invasive role played by extracellular vesicles (EVs), a heterogeneous group of cell-released membranous structures containing various bioactive cargoes, which can be transferred from donor to recipient cells.

**Methods:**

Extracellular vesicles isolated from patient-derived GBM cell lines and surgical aspirates were assessed for their pro-migratory competence by spheroid migration assays, calcium imaging, and PYK2/FAK phosphorylation. Brain invasiveness was investigated in human cortical organoids-based assembloids and in vivo orthotopic xenografts. Extracellular vesicles’ molecular features were specified by multiplex bead-based flow cytometry.

**Results:**

Results unveil a self-sustaining mechanism triggering migration through autocrine release and engagement of a specific population of EVs of large size (L-EVs), isolated from either patient-derived cell lines or surgical aspirates. Large size-EVs act through modulation of calcium transients via Connexin 43-Gap Junctions (Cx43-GJ) and phospho-activation of PYK2. Preincubation with blocking antibodies targeting Cx43 hemichannels demonstrated a dose-dependent inhibition of the L-EV-mediated GBM migration. By exploiting patients’ surgical aspirates, we show that only L-EVs deriving from tumoral cells, and not those with immune origin, promote tumor migration, impacting more prominently the tumoral cells with mesenchymal subtype.

**Conclusions:**

We demonstrate that L-EVs released by GBM cells, but not by the immune cells of the tumor microenvironment, represent a relevant and unique autocrine pro-migratory input for the tumor.

**Key Points**

- Large size-extracellular vesicles (L-EVs) released by glioblastoma (GBM) cells boost tumor migration and brain invasiveness.
- Large size-EV-induced motility depends on their functional, autocrine interaction with GBM cells through Cx43-GJs and calcium signaling modulation.
- At GBM periphery, L-EVs from tumor and stromal cells, but not from immune infiltrating cells, have a prominent impact on tumor invasiveness.
- Large size-EV-induced migration has prominent relevance for GBM cells with mesenchymal subtype, which display notable expression of Cx43.

**Importance of the Study**

The work highlights the importance of glioblastoma (GBM)-derived large subset of extracellular vesicles (referred to as L-EVs) in boosting the invasive behavior of GBM cells. Glioblastoma is renowned for its aggressive infiltration of surrounding brain tissue, leading to recurrence and adverse prognosis. By isolating and characterizing EVs from patient-derived GBM cells and patient surgical aspirates, we found that GBM cells can autonomously boost their own invasiveness through the autocrine release and engagement of L-EVs. Large size-EV autocrine interaction with GBM cells is based on the formation of functional Cx43-GJs, calcium signaling regulation, and PYK2 activation. Data on L-EVs derived from surgical aspirates highlights their potential as indicators of GBM aggressiveness, which is particularly prominent at the periphery of the tumor. Our demonstrations provide a key contribution to clarifying the complexity of GBM invasiveness and identifying common molecular targets to counteract GBM tumor dissemination and progression.

Glioblastoma (GBM) is the most common and malignant primary brain tumor.^1^ More than 2 decades of intensive research have led to only modest improvements in life expectancy.^2^ Besides the elevated heterogeneity across the tumor tissue,^3^ the dismal prognosis is mainly contributed by the wide dissemination of tumor cells that precludes a complete surgical removal and drives tumor recurrence.^4^

Recent research into the mechanisms behind GBM cell motility has shown that GBM-invading cells are spatially enriched in the tumor rim, coinciding with the infiltration zone,^5^ and are regulated by environmental signals, including sensing migratory cues, reorganization of actin cytoskeleton, and interaction with the extracellular matrix.^6^

The dynamic adaptation of GBM cells to environmental conditions contributes to tumor cell survival and resistance to therapy^7^ as well as to tumor invasiveness and progression.^8^ This adaptation induces a shift toward the mesenchymal subtype,^9^ and a high expression of genes related to invasiveness.^10^

Glioblastoma cells adjust to the constantly evolving milieu conditions by establishing intricate tumor-to-tumor and neuro/immune-to-tumor networks, which exploit paracrine mechanisms^11,12^ directing cell-to-cell interaction via gap junctions (GJs),^13^ which support tumor initiation, growth, and dissemination.^5^

In the last years, extracellular vesicles (EVs) have emerged as a sophisticated way of communication among cells, especially in the context of cancer. Extracellular vesicles are a heterogeneous group of cell-released membranous structures, either generated by the outward budding and fission of the plasma membrane, or derived by the inward budding of the endosomal membrane, resulting in the formation of multivesicular bodies.^14^ These 2 mechanisms produce different subtypes of vesicles diverging for size and cargo composition: microvesicles or medium/large vesicles (100–1000 nm) and exosomes or small vesicles (30–150 nm). In both cases, EVs contain a wide variety of bioactive cargoes, which can be transferred to other cells causing phenotypic changes.^15^

Tumor cells, including GBM, produce a significant amount of EVs.^16^ Extracellular vesicles are thought to represent a functional bridge between cells in the GBM context,^17^ promoting angiogenesis,^18^ immunological tolerance,^19^ extracellular matrix (ECM) remodeling,^20^ and shift of brain resident cells into tumor-supportive phenotypes.^21^

By exploiting EVs derived from patient-derived GBM, we demonstrate an autocrine mechanism through which large vesicles enhance the migratory potential of GBM cells. Glioblastoma-derived large size (L)-EVs act through Connexin 43 (Cx43)-GJs, modulating calcium transients and triggering cell migration. Notably, GBM cells bearing mesenchymal transcriptional polarization and expressing higher levels of Cx43 show enhanced responsiveness to L-EV exposure.

## Methods

### Patient-Derived GBM Cell Lines

Glioblastoma cell lines were obtained from surgical specimens of consenting male patients undergoing surgery for brain tumor removal at the Neurosurgery unit of Humanitas Research Hospital, Italy.

Bulk resection samples and tissue fragments in cavitron ultrasonic surgical aspirator (CUSA) washing were quickly dissected mechanically and enzymatically. Further details are given in Supplementary Materials.

### EV Isolation

Extracellular vesicles were isolated by differential centrifugations protocol from cell line conditioned supernatant without growth factors during 24 h of culture. Further details are given in the Supplementary Materials.

### Spheroid Migration Assay

Depending on the cell line, spheroids were generated by culturing 10 000–12 000 cells in the absence of fibroblast growth factor and epidermal growth factor in low attachment round bottom 96-well plates (Sigma). After 4 days, spheroids sized 100–300 μm were transferred on flat-bottom 96-well plates coated with 5 μg/mL of fibronectin (Sigma) and 1 μg/ml of Collagen type I (Sigma).

### Enrichment/Depletion of CD45-Positive EVs

To separate CD45-positive EVs from CD45-negative EVs 2 different commercial kits were combined: (a) microbeads CD45^+^, specific for the selection of CD45 positive cells (Miltenyi) (130-118-780); (b) micro-columns specific for EVs isolation from body fluids (Miltenyi) (130-110-905). Further details are given in the Supplementary Materials.

### Assessment of Multiplex Beads-Based Flow Cytometry Assay

This technique involves the analysis of EVs from surgical aspirates using a bead-based multiplex assay combined with flow cytometry (Multiplex Beads-Based Flow Cytometry Assay [MACSPlex] EVs Kit, human, Miltenyi Biotec). Extracellular vesicle samples were incubated with a set of 37 antibody-coated beads, each targeting different markers. After counterstaining with detection antibodies, the samples underwent flow cytometry to evaluate the expression of these markers, with results corrected for background noise by subtracting values from non-EV negative controls. Further details are given in the Supplementary Materials.

### Orthotopic Xenograft Mouse Model

All experiments in vivo were conducted with the approval of the Italian Ministry of Health. Mice were housed in specific pathogen free animal facility with free access to food and water. 4 × 10^4^ ICH27-PBZ cells transduced with a CMV.Luciferase.ires.eGFP lentivector were stereotactically implanted into the right striatum (x = 2.5, y = −1, z = −3) of 1-month-old CD1 nude male mice (Charles River Laboratories). Large size-EVs administration protocol began when tumor bioluminescence intensity (BLI) reached 104 p/s/cm^2^/sr normalized radiance as measured by IVIS II Imaging (Caliper Life Sciences-PerkinElmer) at approximately day 35 after cell transplantation. Mice were homogeneously randomized in the control group (treated with phosphate buffered saline 1X, *n* = 4) and CUSA-derived CD45-negative L-EVS treated group (*n* = 4). Animals received treatment every 48 h via intranasal administration (1 × 10^9^ L-EVs/d suspended in 10 μL of PBS 1X or saline in equal volume) for a total of 6 administrations. Large size-EVs were derived from CUSA obtained from a single patient. Mice were sacrificed when BLI signal reached the range 10^6^ p/s/cm^2^/sr normalized radiance (day 55 after post-transplantation).

### Brain Tissue and GLICOX Processing

Mice were anesthetized with an intraperitoneal injection of ketamine (100 mg/Kg) and xylazin (10 mg/Kg) mixture, then transcardially perfused with 20 mL of PBS. Free-floating coronal cryosections of brain tissue (10 μm) and GLICOX (12 μm) were incubated with primary and secondary antibodies (for detail see Supplementary Methods).

### Statistics

Statistical analysis was generated with GraphPad Prism software (v10) and the number of technical (*n*) and biological replicates (*N*) are indicated for each experiment. Outliers samples, if present, were removed following the Grubbs test (α = 0.05; http://graphpad.com/quickcalcs/Grubbs1.cfm). Pearson distance measurement method was applied for Heatmap clustering between L-EVs and S-EVs. Heatmaps were created using heatmapper online tool (http://www.heatmapper.ca/expression/).

## Results

### Large EVs Boost GBM Cell Migration

Extracellular vesicles spontaneous release was evaluated in 3 cell lines established from GBM patients (IDH-wt; WHO grade IV) subjected to surgical resection at Humanitas Research Hospital (ICH1, ICH2, and ICH3). Analysis of the expression profile of a curated set of proneural (PN) and mesenchymal (MES) genes allowed the classification of ICH1, ICH2, and ICH3 as predominantly mesenchymal (Supplementary Figure 1A).

**Figure 1.**
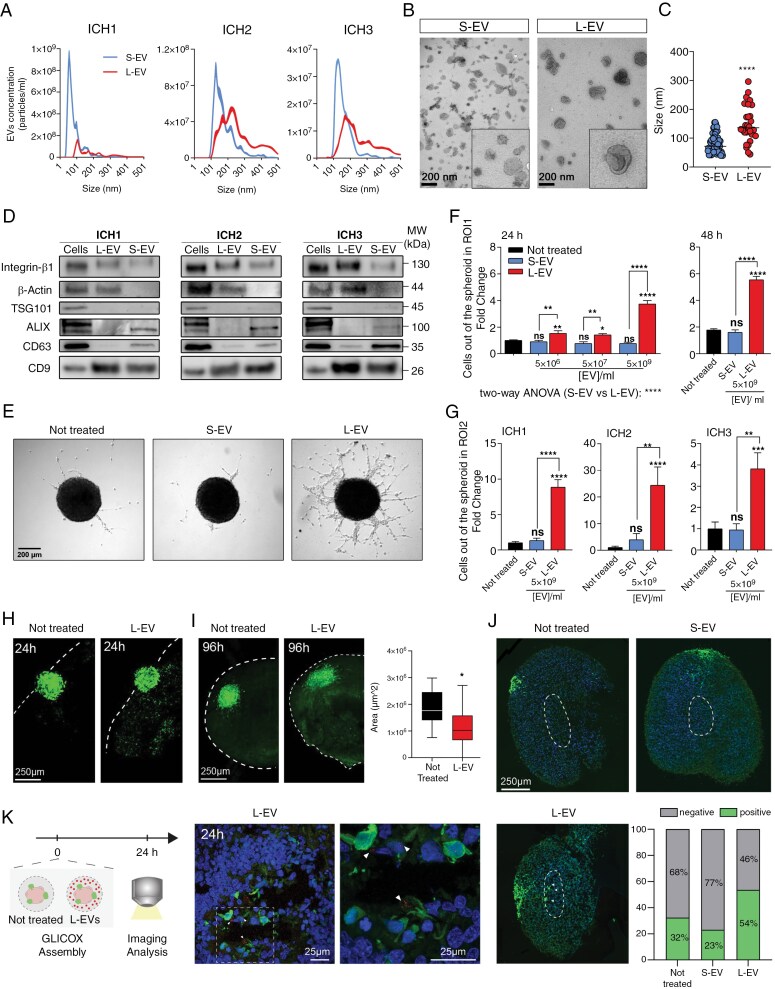
Characterization of extracellular vesicles (EVs) derived from glioblastoma (GBM) lines and their involvement in cell spreading and motility. (A) NTA analysis of EVs isolated from 1 × 10^6^ from three independent cell lines; ICH1 (S-EV *N* = 10, L-EV *N* = 14), ICH2 (S-EV *N* = 7, L-EV *N* = 6), ICH3 (S-EV *N* = 6, L-EV *N* = 8). (B) Representative images of EVs obtained by TEM after negative staining. Scale bar: 200 nm; Image magnification size: 470 × 490 nm. (C) EVs size distribution by TEM analysis. Mann–Whitney test: ****P < .0001. (D) Representative Western blot showing the presence of biogenic EVs markers in cells, L-EV and S-EV lysates. (E) Representative micrograph of the spheroid migration assay after 48 h of a single administration of EVs or in not treated condition. (F) Relative number of cells migrating out of the neurospheres in ICH1 line after 24 and 48 h from a single EVs administration at different concentration. Not treated *N* = 5 (*n* = 36), S-EV *N* = 2 (*n* = 37), L-EV *N* = 3 (*n* = 63). Statistics were generated using two-way ANOVA Bonferroni’s multiple comparison test. L-EV 5 × 10^6^ versus S-EV 5 × 10^6^ **P = .0040, L-EV 5 × 10^6^ versus not treated **P = .0027; L-EV 5 × 10^7^ versus S-EV 5 × 10^7^ **P = .0042, L-EV 5 × 10^7^ versus not treated *P = .0212; L-EV 5 × 10^9^ versus S-EV 5 × 10^9^ ****P < .0001, L-EV 5 × 10^9^ vs not treated ****P < .0001. Relative cell count analysis was performed on neurospheres incubated with 5 × 10^9^ EVs for 48 h. Untreated *N* = 5 (*n* = 36), S-EV *N* = 2 (*n* = 10), L-EV *N* = 3 (*n* = 17). One-way ANOVA with Tukey correction; ****P < .0001. (G) Relative cell count in ROI2 after 24 h from a single administration of EVs: ICH1 untreated *N* = 5 (*n* = 36), S-EV *N* = 2 (*n* = 10), L-EV *N* = 3 (*n* = 16); ICH2 not treated *N* = 2 (*n* = 14), S-EV *N* = 1 (*n* = 4), L-EV *N* = 1 (*n* = 5); ICH3 not treated *N* = 2 (*n* = 26), S-EV *N* = 2 (*n* = 11), L-EV *N* = 3 (*n* = 21). One-way ANOVA multiple comparisons: ICH1: L-EV versus not treated ****P < .0001, L-EV versus S-EV ****P < .0001; ICH2 L-EV versus not treated ***P = .003; ICH3 L-EV versus not treated ***P = .0002, L-EV versus S-EV **P = .0016. All graphs represent mean ± standard error (SE) and they were obtained with cumulative data normalizing on not treated conditions at 24 h. (H) Representative confocal images of whole GLICOX assembloid showing enhanced invasion in L-EV-treated GLICOX compared to untreated after 24 h. (I-left) Representative images of the not treated and L-EVs GLICOX at 96 h showing flattening of the GBM spheroids upon L-EVs treatment. Scale bar, 250 µm. (I-right) Quantification of GBM spheroid area in not treated (*N* = 6 GLICOX, 11 GBM spheroids quantified, 2 independent batches) and L-EVs (*N* = 6 GLICOX, 15 GBM spheroids quantified, 2 independent batches) shows a significant decrease in spheroid area upon L-EVs treatment. Unpaired *t* test with Welch’s correction, *P = .0223. (J) Representative section images of the not treated, S-EV and L-EVs GLICOX. Picture shows increased infiltration of the GBM spheroids upon L-EVs treatment toward the core of the organoid. Scale bar, 250 µm. Quantification of the number of positive section presenting cells in the core of the organoid versus negative sections for not treated, and upon S-EV and L-EV-treatment (not treated: *N* = 3 GLICOX, 360 sections; L-EV: *N* = 3 GLICOX, 216 sections, S-EV: *N* = 1, 176 section). Chi-square statistic with Yates correction in the comparison of not treated versus L-EVs is 8.9963; *P* = .002705, significant at *P* < .05. (K) Analysis of RFP-labeled L-EVs after 24 h of treatment. Schematic representation of the experimental procedure. Confocal images showing dense clusters of RFP-labeled L-EVs localized within GFP-positive GBM cells that have migrated into the assembloid (specific clusters are indicated by white arrows to facilitate interpretation). Magnification of the image is provided. For a better visualization, images of panel K are provided in Supplementary Figure 2G.

The conditioned media from each GBM cell line was collected after 24 h of cell culture. Two fractions of EVs were isolated by exploiting their diverse sedimentation rate (up to 13.000 g or up to 110.000 g) according to MISEV2023 guidelines.^22^ For all the tested lines, size distribution detected by nanoparticle tracking analysis (NTA) confirmed the isolation of 2 distinct fractions enriched with vesicle subsets having median size below or above 200 nm (Figure 1A, Table 1). Accordingly, from now on, these 2 EV subsets will be referred to as S-EVs and L-EVs. The dimensional difference was further confirmed through transmission electron microscopy (TEM) upon negative staining (Figure 1B), showing the isolation of 2 heterogeneous EV populations with distinct sizes; the smaller EVs ranging from 40 to 160 nm and the larger ones reaching diameters up to 280 nm (Figure 1C and Supplementary Figure 1B). Smaller vesicles appeared round-shaped and electron-lucent (Figure 1B, left panel), whereas larger EVs exhibited the collapsed cup-shaped morphology and appeared more electron dense (Figure 1B, right panel). During the sample preparation for conventional TEM techniques, the EVs membrane may collapse, resulting in a cup-shaped morphology.^23^ This phenomenon is related to EVs membrane mechanical stiffness detailed in size, lipid, and protein composition, and it represents one of the important properties that can distinguish different classes of EVs.^24^

**Table 1.** NTA Size Distribution and Quantification of Small and Large Vesicles Derived From GBM Lines (ICH1-ICH2-ICH3) in 24 h. Extracellular Vesicles (EVs) Quantifications are Expressed as Median Value ± SE

Cell line	L-EVs		S-EVs	
	Size (nm)	Concentration (particles/mL)	Size (nm)	Concentration (particles/mL)
ICH1	199.00 ± 5.44	(0.67 ± 0.17) × 10⁹	153.80 ± 10.16	(10.00 ± 2.75) × 10⁹
ICH2	212.50 ± 9.99	(2.20 ± 5.70) × 10⁹	153.88 ± 5.76	(9.58 ± 1.09) × 10⁹
ICH3	211.75 ± 12.70	(0.65 ± 0.11) × 10⁹	60.07 ± 4.09	(14.28 ± 0.11) × 10⁹

In accordance with their intraluminal origin, small EVs displayed an enrichment of CD63 tetraspanin and of the endosomal sorting complex (ESCRT) proteins Alix and TSG101 (Figure 1D).^25^ Conversely, the enrichment of proteins associated with the plasma membrane such as integrin β1 (ITGB1) and β-actin indicates that the isolated large EVs originate from the cell outer membrane.^26^ Consistently with its membrane localization and overexpression in GBM stem cells, CD9 tetraspanin was expressed in both S-EV and L-EVs.^27^

Glioblastoma is characterized by a rapid and highly infiltrative growth, being its invasive nature the main source of recurrence after surgery.^28^ To explore whether the migratory potential of the patient-derived GBM lines could be promoted by EVs, an in vitro migration test was set up using GBM cell spheroids. Glioma spheres were challenged with EVs (Figure 1E), and cells moving outside the spheroid were scored in terms of (a) count of cells out of the neurosphere within a fixed field defined as region of interest 1 (ROI1, Figure 1F) and (b) count of cell migrating beyond a line equal to spheroid diameter (ROI2; Figure. 1F, mean distance higher than 360 µm, Supplementary Figure 1C). To prevent cell replication that could alter the quantification of cell mobility, all migration experiments were carried out employing medium without growth factors. The lack of cell division in this experimental condition was verified by MTT and neurosphere formation assays (Supplementary Figure 1E).

Spheroids were challenged with autologous L-EVs or S-EVs, at concentrations of 5 × 10^6^, 5 × 10^7^, or 5 × 10^9^/mL for 24 or 48 h. While S-EVs exerted no effect on GBM migratory activity, L-EVs triggered a dose-dependent enhancement of the number of cells migrating out of the neurosphere, which was particularly evident when spheroids were treated at 5 × 10^9^/mL dose (Figure 1G, ICH1; Supplementary Figure 1F, ICH2 and ICH3). The number of cells moving outside the spheroid core (ROI1) further increased over time when the incubation was extended to 48 h, with S-EVs producing again no effect (Figure 1G, ICH1; Supplementary Figure 1G, ICH2 and ICH3). The activity of L-EVs on GBM cells was also evident upon quantification of cells migrating over a long distance in ROI2 (Supplementary Figure 1D).

To summarize, L-EVs sustain GBM migratory capacity increasing the number of cells moving out of the spheroid and covering longer distance within the same time frame.

### L-EVs Boost GBM Cell Invasion in a 3D Organoid

Tumoral cell spreading in GBM encompasses cellular migration, represented by the intrinsic ability to move in free space, and infiltration, which requires active microenvironment remodeling by the cells penetrating the surrounding tissue. To assess whether the pro-migratory effect of L-EVs also had implications for cell invasion dynamics, we exploited 3-dimensional human GLICOX assembloids. As self-organizing 3D culture systems derived from human induced pluripotent stem cells (hIPSCs), cortical organoids display the basic organization of the human cortex.^29^ Upon combining 2-month-old cortical organoids with green fluorescent protein (GFP) -transduced GBM spheroids, we generated and monitored the cortical-tumor assembloids (Supplementary Figure 2A), followed by longitudinal confocal imagining and quantitative analysis by IMARIS (Supplementary Figure 2B). This allowed us to follow the dynamic changes in GBM spheroid morphology and parenchyma invasion along the treatment.

After 96 h in culture, a significant increase in GBM spheroid area was observed (Supplementary Figure 2C), supporting that GBM cells penetrate the brain organoid parenchyma. Analysis of GLICOX sections confirmed the intrinsic ability of GBM cells to migrate within the organoid and invade it (Supplementary Figure 2D), although, within this time frame we rarely observed GFP-positive cells reaching the most inner core of the organoids. We next investigated whether L-EVs could induce changes in GBM cell invasion, by exposing GLICOX assembloids to 5 × 10^9^ L-EVs/mL, carrying along the S-EVs as control (5 × 10^9^ S-EVs/mL) (DIV0; Supplementary Figure 2E). Already after 24 h from L-EV administration, we observed a more diffuse invasion of GFP-positive cells into the GLICOX (Figure 1H). Quantitative analysis of the GBM spheroid area after 96 h showed that L-EV treatment decreases the tumor cell area on the organoid surface (Figure 1I). In addition, L-EV but not S-EV treatment increased the number of cells reaching the center of the organoid (Figure 1J), suggesting that L-EVs selectively boost the invasive behavior of GBM cells. To follow the L-EV localization into the GLICOX, we exposed assembloids to stably labeled vesicles, isolated upon ultracentrifugation from red fluorescent protein (RFP) -expressing tumor cells. We first observed the presence of RFP-L-EVs in treated GLICOX (Figure 1K, Supplementary Figure 2F), where clusters of vesicles were primarily found close to GFP-expressing cancer cells within the assembloids (Figure 1K). These data demonstrate that L-EVs enhance GBM penetration even in a human 3D culture system, supporting a role for L-EVs not only in GBM cell migration but also in cell infiltration.

### Tumor Derived L-EVs Isolated From Patient Surgical Aspirate Enhance Cell Migration

**Figure 2.**
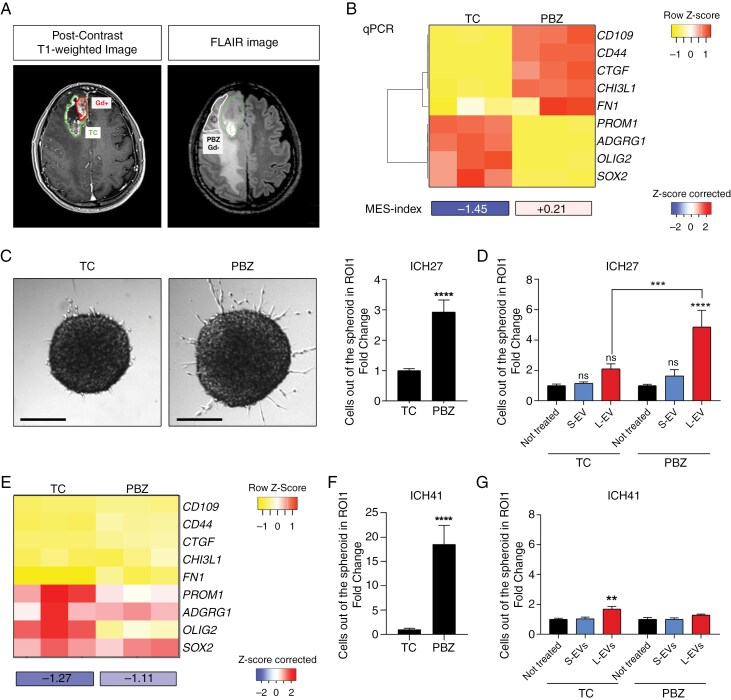
L-EVs, but not S-EVs, isolated from cell lines derived from different tumor regions show different ability to enhance cell migration. (A) Preoperative magnetic resonance imaging of patient 27. (left) Gadolinium (Gd) enhanced T1 weighted images, (right) the respective nonenhancing FLAIR-zone visible in T2 segmentation. Tumor core (TC), area from which ICH27-TC (Gd+) and ICH27-PBZ (Gd−) were isolated are indicated. (B) (Upper panel) Heatmap of the transcriptional profile of matched ICH27-TC and ICH27-PBZ cell lines performed using q-PCR (*N* = 3). (Lower panel) Calculated mesenchymal score for both cell lines. (C) (Left) Representative image of matched ICH27-TC versus ICH27-PBZ neurospheres (scale bar: 200 nm). (Right) Migration analysis of ICH27-TC and ICH27-PBZ spheroids after 24 h of culture under basal conditions. Results are reported as fold increase of migrating PBZ cells on the TC counterpart (*N* = 3) (*n* = 33). Unpaired *t* test ****P < .0001. (D) Relative cell count of ICH27-TC and ICH27-PBZ spheroids treated with 1.5 × 10^8^ of autologous EVs for 24 h. ICH27-TC not treated *N* = 2 (*n* = 10), S-EV *N* = 2 (*n* = 10), L-EV *N* = 2 (*n* = 8); ICH27-PBZ not treated *N* = 2 (*n* = 9), S-EV *N* = 2 (*n* = 9), L-EV *N* = 2 (*n* = 9). Two-way ANOVA original FDR method of Benjamini and Hochberg multiple comparison test. ICH27-PBZ: not treated versus L-EV ****P < .0001, PBZ L-EV versus TC L-EV ***P = .0002. Data are normalized to the not treated sample and graphs were generated on cumulative data. Histograms and bars display mean ± SE. (E) (Upper panel) Heatmap of the transcriptional profile of matched ICH41-TC and ICH41-PBZ cell lines performed using q-PCR (*N* = 3). (Lower panel) Calculated mesenchymal score for both cell lines. (F) Migration analysis of ICH41-TC and ICH41-PBZ spheroids after 24 h of culture under basal conditions. Results are reported as fold increase of migrating PBZ cells compared to the TC counterpart (*N* = 3) (*n* = 33). (G) Relative cell counts of ICH41-TC and ICH41-PBZ spheroids treated with 1.5 × 10^8^ of autologous EVs for 24 h. ICH41-TC not treated *N* = 2 (*n* = 10), S-EV *N* = 2 (*n* = 10), L-EV *N* = 2 (*n* = 10); ICH41-PBZ not treated *N* = 2 (*n* = 9), S-EV *N* = 2 (*n* = 8), L-EV *N* = 2 (*n* = 6). Two-way ANOVA original FDR method of Benjamini and Hochberg multiple comparison test. ICH41-TC: not treated versus L-EV **P = .0014, Data are normalized to the not treated sample and graphs were generated on cumulative data. Histograms and bars display mean ± SE.

Given the clinical relevance of the tumor cells infiltrating healthy brain parenchyma^30^ L-EV pro-migratory potential was investigated in cells isolated from the peri-tumoral area surrounding the contrast-enhancing components and identified by the hyperintense signal on Fluid Attenuated Inversion Recovery (FLAIR) images (FLAIR-zone). Two different cell lines were generated from the same patient (patient 27) (Figure 2A): (a) a cell line identified as Peritumoral Brain Zone (PBZ) (ICH27-PBZ) established from the brain parenchyma showing hyperintensity on FLAIR images obtained through ultrasonic tissue fragmentation which allows to capture residual GBM cells beyond the bulk resection margins; (b) a second cell line isolated in parallel from the Gadolinium (Gd)-enhancing portion of the lesion of the tumor core (ICH27-TC), visible on postcontrast T1-weighted images. Subtype characterization of the established lines revealed ICH27-PBZ as having a predominant MES signature, and ICH27-TC as having a PN signature (Figure 2B).

The constitutive migratory capacity of the 2 established lines was evaluated through spheroid assays in the absence of EVs. Interestingly, ICH27-PBZ and ICH27-TC showed different innate migratory phenotypes, potentially mirroring the features typical of the tumor zone from which they were isolated^5^ with ICH27-PBZ showing a more prominent migratory phenotype compared to ICH27-TC (Figure 2C). Large size-EVs and S-EVs generated from each cell line (Table 2, Supplementary Figure 3A and B) were then used to evaluate their possible migration-enhancing effect. Spheroids challenged with autologous vesicles confirmed that L-EVs, but not S-EVs, triggered remarkable cell migration, which was however more evident for ICH27-PBZ (Figure 2D).

**Table 2.** NTA Size Distribution and Quantification of Small and Large Vesicles Derived From the ICH27-PBZ Cell Line in 24 h. Extracellular Vesicles (EVs) Quantification is Expressed as Median Value ± SE

Cell line	L-EVs		S-EVs	
	Size (nm)	Concentration (particles/mL)	Size (nm)	Concentration (particles/mL)
ICH27-PBZ	208.90 ± 68.65	(1.26 ± 0.08) × 10^10^	133.40 ± 21.75	(1.19 ± 1.12) × 10^10^

Among the cell lines tested, only the ICH27-TC did not show a significant increase in cell migration following exposure to L-EV. Of note, this was the only cell line showing a proneural subtype, while the remaining four were characterized by mesenchymal polarization, opening the possibility that the GBM transcriptional heterogeneity might influence the migratory response induced by L-EVs. The inclusion in the analysis of additional paired cell lines (ICH41-TC and ICH41-PBZ) derived from patient 41 and both displaying a proneural phenotype (Figure 2E) revealed that, as for ICH27-PBZ, ICH41-PBZ showed a more prominent migratory phenotype compared to ICH41-TC (Figure 2F). Nevertheless, like proneural ICH27-TC, also ICH41-TC and ICH41-PBZ reported limited migration enhancement following L-EV treatment (Figure 2G), supporting the possibility that mesenchymal cells may be more responsive to L-EV-induced pro-migratory stimuli (Supplementary Figure 3C).

**Figure 3.**
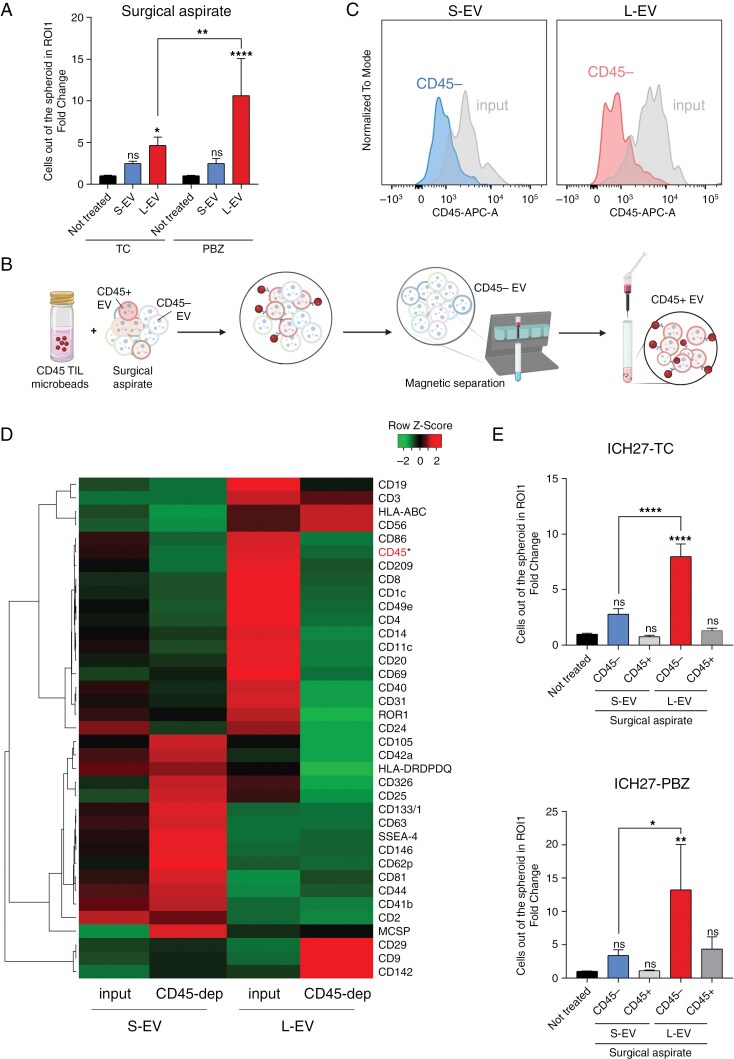
Tumor-derived L-EVs isolated from surgical aspirate induce GBM cells migration. (A) Spheroid migration assay was performed on ICH27 spheroids treated with EVs (1.5 × 10^8^/mL) isolated from autologous surgical aspirate. Cells moving out from the core sphere were counted after 24 h of incubation. ICH27-TC not treated *N* = 3 (*n* = 16), S-EV *N* = 3 (*n* = 18), L-EV *N* = 3 (*n* = 16); ICH27-PBZ not treated *N* = 3 (*n* = 15), S-EV *N* = 3 (*n* = 16), L-EV *N* = 3 (*n* = 9). Two-way ANOVA original FDR method of Benjamini and Hochberg multiple comparison test: ICH27-TC not treated versus L-EV *P = .0363; ICH27-PBZ not treated versus L-EV ****P < .0001; PBZ L-EV versus TC L-EV ****P < .0001. (B) Schematic EV enrichment protocol using CD45 Microbeads and EV Micro-Columns. (C) CD45 MFI obtained by FACS analysis following a bead-based depletion of CD45-positive EVs from GBM patients’ 27 surgical aspirates. (D) Heatmap comparison of MFI marker samples obtained using the MACSplex multiplex flow cytometry assay. Samples analyzed before depletion (input, left) and after depletion (CD45-negative, right) were S-EVs (10^9^) and L-EVs (10^8^) isolated from the surgical aspirate of patients 27. Data are shown as *Z*-score and scaled by row. Clustering method: centroid linkage, Pearson distance. (E) Migration assay of ICH27 spheroids treated with CD45 enriched/depleted EVs. Spheres were challenged with an EV dose of 1.5 × 10^8^/mL for 24 h. ICH27-TC (upper panel) not treated *N* = 3 (*n* = 16), S-EV CD45-negative *N* = 3 (*n* = 17), S-EV CD45-positive *N* = 3 (*n* = 16), L-EV CD45-negative *N* = 3 (*n* = 18), L-EV CD45-positive *N* = 3 (*n* = 18). ICH27-PBZ (lower panel) not treated *N* = 3 (*n* = 16), S-EV CD45-negative *N* = 3 (*n* = 18), S-EV CD45-positive *N* = 3 (*n* = 17), L-EV CD45-negative *N* = 3 (*n* = 11), L-EV CD45-positive *N* = 3 (*n* = 15). One-way ANOVA Tukey multiple comparison test. ICH27-TC: not treated versus L-EV CD45-negative ****P < .0001, S-EV CD45-negative versus L-EV CD45-negative ****P < .0001. ICH27-PBZ: not treated versus L-EVs CD45-negative **P = .0089, S-EV CD45-negative versus L-EV CD45-negative *P = .0488. All migration tests were normalized on not treated condition and graphs were generated with cumulative data. Histograms and bars display mean ± SE.

To further investigate the relevance of PBZ-EVs to boost tumor cell invading capacity, we isolated L-EVs from the periphery of tumors, exploiting the fluid collected during surgery through the CUSA (Supplementary Figure 3D). Extracellular vesicles isolated from patient 27 surgical aspirate displayed mean concentrations of 10^11^ (Table 3), in line with the concentrations observed in the aspirates from a cohort of 6 additional patients (Supplementary Figure 3E), indicating that the concentrations we used in in vitro studies were not artificially excessive. Large size-EVs from surgical aspirate of patient 27 were demonstrated to be even more powerful in inducing cell migration, compared to L-EVs isolated from cell line supernatants (Figure 3A). Similarly to previous results, S-EVs demonstrated limited effect. Conversely, L-EVs exerted a significant pro-migratory activity that was more prominent toward ICH27-PBZ than ICH27-TC.

**Table 3.** NTA Size Distribution and Quantification of EVs Isolated From Patient 27 Surgical Aspirate, Whole Set and CD45-Negative Extracellular Vesicles (EVs). EV Quantification is Expressed as Median Value ± SE

Surgical aspirate	L-EVs		S-EVs	
	Size (nm)	Concentration (particles/mL)	Size (nm)	Concentration (particles/mL)
Whole set	276.00 ± 56.17	(3.49 ± 2.10) × 10^11^	205.80 ± 30.28	(2.82 ± 1.79) × 10^11^
CD45^−^	235.30 ± 74.01	(7.35 ± 5.28) × 10^10^	167.40 ± 9.83	(1.15 ± 1.08) × 10^11^

Given the abundance of the immune infiltrate in GBM^31^ and its competence to secrete EVs,^32^ it is expected that the surgical aspirate contains a mixture of EVs released by both tumoral and nontumoral cells. This assumption was confirmed by a multiplex beads-based flow cytometry analysis, which revealed the abundance of markers associated with immune cells and a very low amount of CD62p/P-selectin, CD105 and CD31/PECAM-1 endothelial markers (Supplementary Figure 3F, for the gating strategy used see Supplementary Figure 3G). To distinguish the cell origin of L-EVs exerting a pro-migratory activity, we settled a purification protocol to segregate EVs on the basis of CD45 immune marker expression (Figure 3B). By this approach, we were able to isolate from the surgical aspirate both CD45-positive EVs (CD45^+^) released by immune cells and CD45-negative EVs (CD45^−^) released by tumor and stroma cells (Table 3, Supplementary Figure 3H). The specificity of the isolation was confirmed by the multiplex beads-based flow cytometry analysis, which revealed (a) the efficacy of CD45 depletion in relation to input material (Figure 3C), (b) the prominent depletion of immune lineage markers (CD3, CD4, CD8, CD11c, CD14, CD19, CD20, and CD1c) in CD45^−^ EVs (Figure 3D), and (c) the high enrichment, in CD45^−^ L-EVs, of the tumor-associated markers CD29 and CD142^33–35^ as well as of CD56 which, despite being associated with the immune compartment, also has a biological role in different tumors.^36^ These analyses confirmed the effectiveness of the CD45-based separation (Figure 3D).

Notably, the results obtained by the migration assay performed with either ICH27-TC or ICH27-PBZ spheroids revealed the absence of any remarkable pro-migratory effect by CD45-positive EVs (Figure 3E). On the contrary, a significant migration boost was observed upon the treatment with CD45-negative EVs, further indicating that EVs generated by tumor and stroma cells play a key role in tumor invasion (Figure 3E). Again, the pro-migratory effect of CD45-negative EVs was limited to L-EVs, in line with their specific enrichment with tumor markers associated with cancer aggressive behavior.

### L-EVs Support GBM Cell Infiltration of the Brain Parenchyma

**Figure 4.**
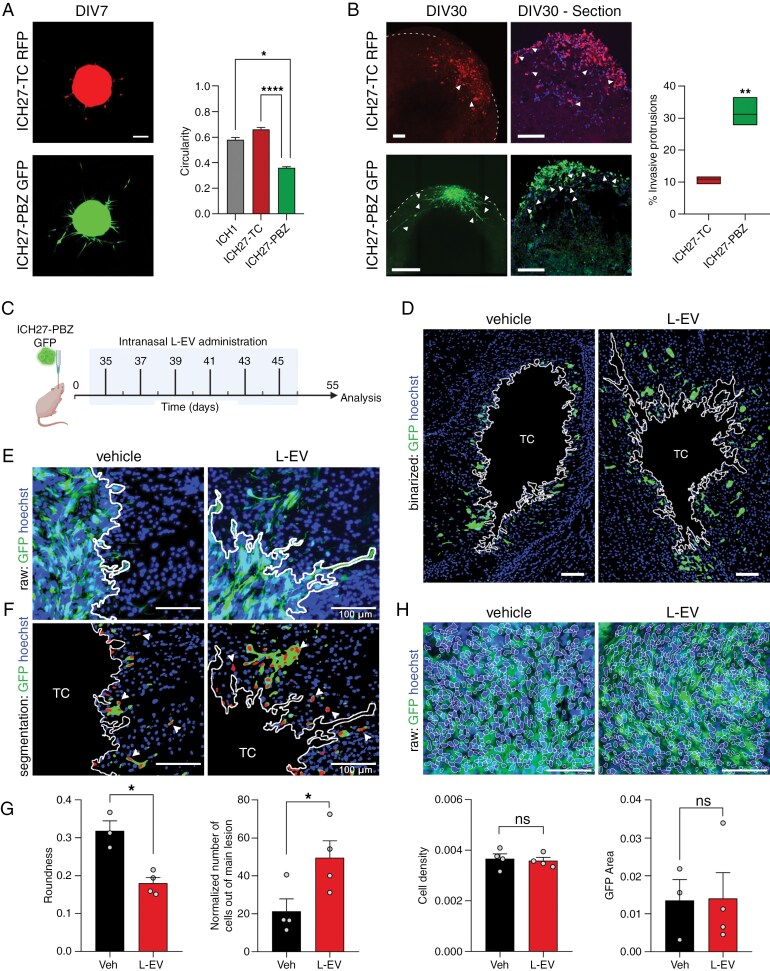
L-EVs increase tumor cell invasion in vivo. (A-left) Representative images of ICH27-TC (RFP-expressing) and ICH27-PBZ (GFP-expressing) spheroids after 7 days. (A-right) Bar graph quantification of the circularity parameter of cells migrating out of the spheroids (*N* = 4). Unpaired *t*-test with Welch’s correction; ****P < .0001; *P = .0118. (B-left) Representative live-images of ICH27-TC (RFP-expressing) and ICH27-PBZ (GFP-expressing) assembloids at DIV30 and relative sections. Examples of invasive protrusions are marked by arrowheads. (B-right) Bar graph quantification of the percentage of invasive protrusions, showing a significantly high number in ICH27-PBZ GLICOX. Invasive protrusions were counted on 3 organoids per condition and the average number of invasive protrusions was calculated across multiple sections (minimum of 2 per organoid). Unpaired *t*-test; *P = .0013. (C) Schematics showing in vivo experimental procedure. (D) Representative binarized image of brain sections exhibiting tumor core region (TC) and surrounding areas in the vehicle (left) and L-EV treated (right) mice. Tumor cells were labeled with antibody against GFP and nuclei counterstained with Hoechst. TC margin was automatically detected and displayed with a solid line. (E) Representative peritumoral zone at higher magnification. (F) Binarized images allowing nuclei segmentation within the GFP-positive area and automatic scoring of cells migrating out of the TC region (examples of the scored nuclei are indicated with arrows). (G) Right: evaluation of tumor rim in terms of roundness: *P < .05. Vehicle *N* = 3 (*n* = 10), L-EV *N* = 4 (*n* = 15). Left: quantification of the migrating cells out of the tumor area normalized by the extension of the TC area: *P < .05. Vehicle *N* = 4 (*n* = 13), L-EV *N* = 4 (*n* = 13). (H) Upper panel: representative images of the central tumor area depicting automatic nuclei recognition (solid lines). Lower panel: quantification of cell density (number of nuclei/central tumor area) and GFP area. Scale bars: 100 µm. Statistical comparison calculated with Welch corrected unpaired *t*-test post Shapiro–Wilk normality test assessment. Vehicle *N* = 4 (*n* = 17), L-EV *N* = 4 (*n* = 26). Histograms and bars display mean ± SE.

To demonstrate the capacity of L-EVs to promote GBM cell migration and invasion in vivo, CD1 nude mice were orthotopically injected with GFP transduced GBM cells. ICH27-PBZ cells were chosen to generate the in vivo model on the basis of their in vitro morphotype both on 2D and 3D settings indicating a higher invading pattern (Figure 4A and B).^37^ In particular, cells migrating out of the ICH27-PBZ spheroids displayed elongated morphology with long branches spreading and lower circularity comparing to ICH27-TC and ICH1 (Figure 4A). Similarly, ICH27-TC (RFP+) and ICH27-PBZ (GFP+) cells in the 3D GLICOX assembloid setting displayed equivalent morphotypes, with ICH27-PBZ cells showing an increased number of invasive protrusions (Figure 4B).

Starting from 4 weeks after intracranial inoculation, mice were treated every 48 h with intranasal administration of 1 × 10^9^ CD45-negative L-EVs freshly derived from a patient surgical aspirate, for a total of 6 deliveries (Figure 4C). Within 2 months, the injected cells generated a localized tumoral lesion with nonnecrotic core and with margins characterized by variable rates of infiltration (Figure 4D). To quantify the tumor infiltrative capacity, the central tumor outline was evaluated in terms of roundness (R). The more jagged and infiltrative the tumor, the lower the R parameter will be. This approach allowed to objectively describe the tumor infiltrative attitude within each sample. Mice treated with L-EVs showed a higher tumor invasion compared to the control group as indicated by the lower R value (Figure 4E and G, R_CTRL_ = 0.32, R_L-EV_ = 0.18, *P*-value = .017). In addition, the number of cells detaching from the central lesion and invading the brain parenchyma was scored to further quantify the gained invading capacity of the tumor after L-EVs treatment (Figure 4F and G). Notably, the increased invasive potential was not paralleled by a different GFP area or cell density within the central lesion (Figure 4H).

These results confirm our previous in vitro observations, demonstrating the contribution of L-EVs in promoting a pro-migratory behavior in GBM.

### L-EVs Promotion of Cell Migration Requires Connexin-43 Hemichannels

We next aimed to gain more insights into the mechanisms by which L-EVs enhance GBM migration and invasion. We focused our attention on GJ proteins, such as connexins, which provide direct functional communication between adjacent cells and are known to be expressed in GBM cells contributing to the dissemination of cancer stem cells.^38,39^ As transmembrane protein channels, connexin hemichannels have been detected on the surface of L-EVs budding from the plasma membrane.^40–42^

**Figure 5.**
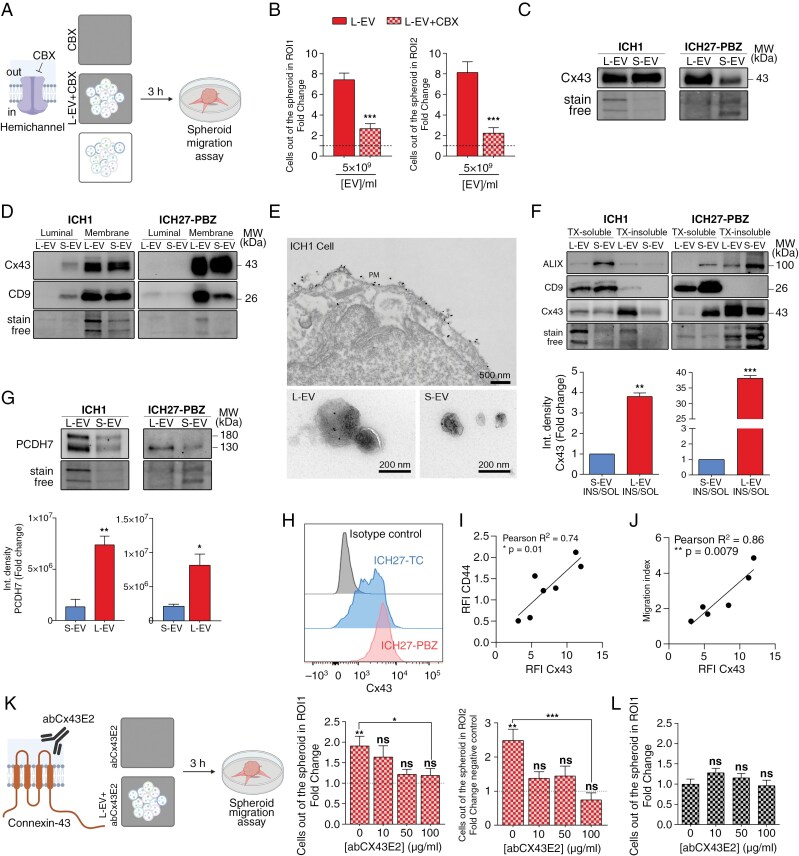
L-EVs increase cell motility by Cx43 gap junctions. (A) Cartoon of the experimental design. Migration assays were performed on neurospheres treated in the presence/absence of L-EV with or without CBX-preincubation (100 uM). (B) Spheroid migration assay 24 h after L-EV treatment with or without CBX preincubation. Data are normalized on the same CBX conditions without L-EV treatment (dashed line, see Supplementary Figure 4A) and histograms depict mean ± SE. Statistic was obtained by unpaired *t* test: ROI 1 L-EV versus L-EV + CBX ***P = .0002, ROI 2 L-EV versus L-EV + CBX ****P = .0005. L-EVs *N* = 1 (*n* = 6), L-EVs CBX *N* = 1 (*n* = 6). (C) Representative immunoblot of Connexin 43 (Cx43) expressed in small and large EVs derived from ICH1 (*N* = 8) and ICH27-PBZ (*N* = 3). (D) Representative Western blot showing Cx43 and CD9 at luminal and membrane level in ICH1 (*N* = 2) and ICH27-PBZ (*N* = 2) derived EVs. (E) Representative images of Cx43 nanogold immunodetection on ICH1 cell and EVs. Reference scale bar is indicated. (F) Detection of triton x-100 soluble and insoluble form of Cx43, Alix, and CD9 at EVs level. Representative blot of ICH1 (*N* = 2) and ICH27-PBZ (*N* = 2) derived EVs. Unpaired *t* test was applied for statistical analysis: **P = .0039, ***P = .0006. Integrated density of L-EVs is normalized on stain free and data are reported normalized on S-EVs chemiluminescent signal. (G) Representative images of immunoblot carried out to detect the amount of protocadherin7 (PCDH7) on ICH1 and ICH27-PBZ derived EVs (*N* = 3). Unpaired *t* test: **P = .0056, *P = .0217. Data are normalized on stain-free chemiluminescence signal. (H) Representative fluorescence intensity at flow cytometry of Cx43 protein detected in ICH27-TC and ICH27-PBZ cells. Isotype control is indicated as reference of negative signal. (I) Correlation analysis between the RFI of mesenchymal marker CD44 and Cx43, across the patient-derived cell lines used in this study. Statistical analysis was performed using Pearson correlation: *R*^2^ = 0.74, *P = 0.01. (J) Correlation analysis between GBM cell line responsiveness to L-EVs (migration index as relative mean cell out of the spheroids after 24 h from L-EV exposure) and Cx43 RFI across the patient-derived cell lines used in this study. Statistical analysis was performed using Pearson correlation: *R*^2^ = 0.86, **P = .0079. (K) Upper panel: cartoon of the experimental design. L-EVs were preincubated with the Cx43-blocking antibody (AbCX43E2) and then used as stimuli for the spheroid migration assay. Bottom: AbCX43E2 blocking antibody dose–response migration assay quantification. L-EVs (5 × 10^9^/mL medium) were preincubated with different concentrations of AbCX43E2 blocking antibody (10, 50, or 100 μg/mL). Not treated *N* = 3 (*n* = 28), L-EVs *N* = 3 (n = 24), L-EVs + AbCX43E2 at 10 μg/mL *N* = 2 (*n* = 16), at 50 μg/mL *N* = 2 (*n* = 15), at 100 μg/mL *N* = 2 (*n* = 12). Statistical analysis was performed with cumulative data using two-way ANOVA Bonferroni’s multiple comparisons test: *P = .0151, **P = .0011, ****P < .0001. Samples were normalized on paired controls treated without L-EVs and with comparable abCX43E2 scalar concentrations (dotted line, see Figure 5L). (L) Spheroid migration assay quantification of the pro-migratory effect of AbCX43E2 alone on neurospheres. The different amounts of Cx43 blocking antibody used during the preincubation are indicated. CTRL *N* = 4 (*n* = 28), AbCX43E2 10 μg/mL *N* = 3 (*n* = 17), AbCX43E2 50 μg/mL *N* = 2 (*n* = 12), AbCX43E2 100 μg/mL *N* = 2 (*n* = 11). The final concentration of blocking antibody on neurospheres varied depending on the EV concentration. All the histograms display mean ± SE and data are normalized on not treated conditions.

To investigate whether functional GJs are formed between L-EV and GBM cells, L-EVs were preincubated with carbenoxolone (CBX), a nonselective channel blocker, and their capacity to promote migration was tested (Figure 5A). In line with a role of GJs in L-EV-mediated GBM cell migration, CBX significantly reduced the L-EVs pro-migratory potential (Figure 5B). Of note, CBX by itself did not influence cell migration (Supplementary Figure 4A). The bulk of GBM cells are organized in intercellular networks supported by direct cell-to-cell contacts achieved through Cx43-GJ formation.^43^ Connexin 43, also known as gap junction protein-α1 (gene name: *GJA1*), is the most predominant GJ protein expressed in the brain.^44^ It is enriched in reactive astrocytes and is one of the most important players in the glia-neuro-vascular crosstalk.^45^ In our model, Cx43 was detected in both S-EV and L-EV (Figure 5C and Supplementary Figure 4B) and is preferentially enriched at the membrane level (Figure 5D), which suggests the potential ability of EV-associated Cx43 to self-assemble in functional connexons. Moreover, by immuno-electron microscopy, we demonstrated the localization of Cx43 on the plasma membrane of GBM cells as well as on vesicles located just outside the cell surface and on isolated EVs (Figure 5E).

To evaluate the ability of Cx43 to assemble into hemichannels, a balanced partitioning between Triton X-100 soluble and insoluble forms^46^ was performed (Figure 5F). The majority of Cx43 in L-EVs was found as insoluble aggregates (Figure 5F), thus suggesting the preponderant assembly of Cx43 into GJ-hemichannels. Conversely, soluble fraction in S-EVs was predominant, thus indicating that Cx43 was not organized in hemichannels. Finally, and consistently with the presence of functional Cx43-hemichannels on the membrane of L-EV but not S-EVs, a significantly higher expression of protocadherin 7 (PCDH7)^47^ was detected in L-EVs compared to S-EVs (Figure 5G and Supplementary Figure 4C).

To further confirm the role of Cx43 in L-EVs promotion of cell migration, we assessed the possible correlation between Cx43 and GBM cell line responsiveness to L-EVs. Given our indication that mesenchymal cell lines are more responsive to L-EV-induced pro-migratory response (Figure 2 and Supplementary Figure 3C), we correlated the expression of *GJA1* with the mesenchymal marker *CD44*, using both single-cell^48^ (Supplementary Figure 4D) and bulk RNA-seq of TCGA-GBM patient samples^49^ (Supplementary Figure 4E) from publicly available datasets. These findings were validated in our sample collection, as indicated by the prominent abundance of Cx43 in mesenchymal GBM cells at flow cytometry analysis (Figure 5H). The relative fluorescence intensity (RFI) of CD44 and Cx43 showed a clear positive correlation (Figure 5I), thereby confirming findings previously established through independent public datasets. Importantly, a significant correlation between Cx43 expression and L-EV-induced cell migration index was also observed, suggesting that responsiveness to L-EVs depends on Cx43 expression (Figure 5J).

To demonstrate whether Cx43 is required for the L-EV-mediated increase of tumor cell migration, a blocking antibody directed against the second extracellular loop region of Cx43 (E2), and able to specifically target Cx43 hemichannels was exploited^50^ (Figure 5K). The E2 blocking antibody has the advantage, compared to other Cx43 blockers,^42,51^ of not interfering with preexisting GJs, while preventing the docking and formation of new connexons,^51,52^ it allows to selectively investigate the role of newly formed GJs upon exposure of GBM to EVs. Preincubation of vesicles with different concentrations of blocking antibody demonstrated a dose-dependent inhibition of the L-EV-mediated GBM migration. Incubation of neurospheres with the Cx43-blocking antibody alone (CTR/untreated) did not affect the pro-migratory potential on GBM cells (Figure 5L). In addition, disruption of L-EVs via subsequent freeze and thaw cycles followed by centrifugation of the membranous fraction^53^ revealed that, although containing Cx43, the L-EV membrane insoluble fraction failed to boost cell migration (Supplementary Figure 4F).

These data indicate that the pro-migratory effect of L-EVs requires the presence of functional Cx43 hemichannels, also pointing to a channel role for Cx43. They also suggest that the reduced capacity of S-EVs to boost GBM migration may result from either the difficulty in establishing effective connexons through Cx43 channels or by S-EV versus L-EV different cargo.

### L-EVs Induce Intracellular Calcium Transients

**Figure 6.**
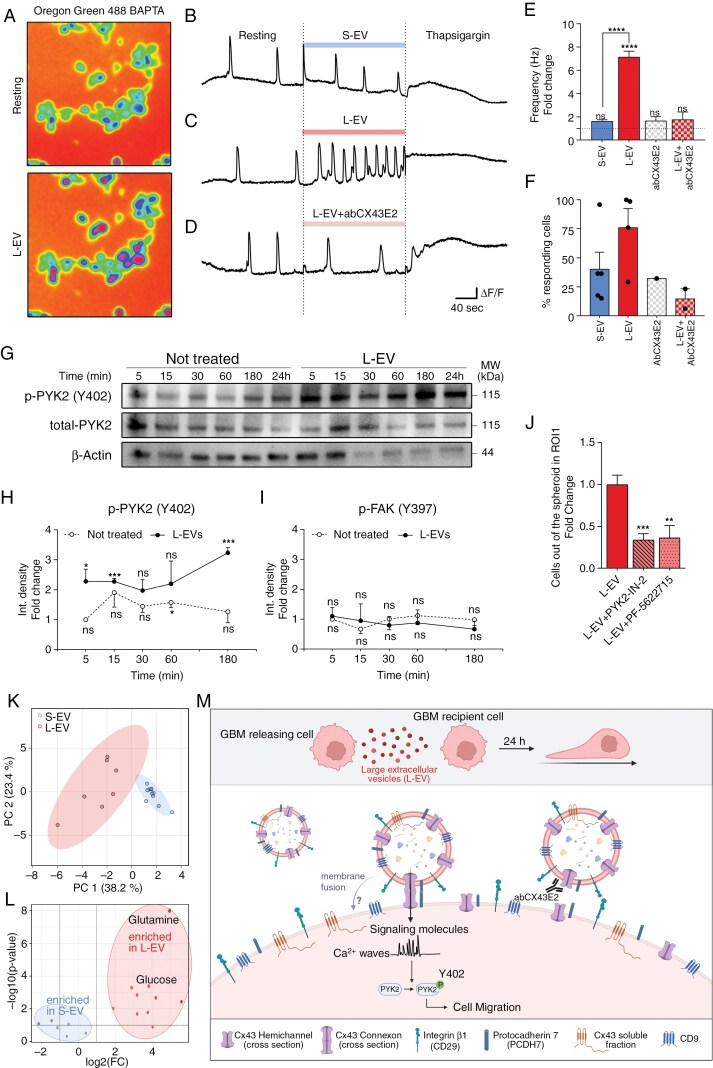
L-EVs trigger cell motility by increasing intracellular calcium transients and PYK2 activation. (A) Representative image of Oregon Green stained cells in basal condition (upper panel) and after L-EV treatment (lower panel). (B–D) Representative traces of single cell calcium imaging recording under resting condition, treatment stimuli, and Thapsigargin (300 s for each part). Treatment stimuli were S-EV (B), L-EVs (C), and L-EVs preincubated with AbCX43E2 (D). (E) Frequency of calcium waves in cells responding to stimuli with respect to resting condition (dashed line). S-EVs *N* = 5 (*n* = 197), L-EVs *N* = 4 (*n* = 195), AbCX43E2 *N* = 1 (*n* = 16), L-EVs preincubated with AbCX43E2 *N* = 2 (*n* = 9). Statistical analysis was performed with cumulative data using ordinary one-way ANOVA Multiple comparisons; L-EVs versus basal ****P < .0001, L-EVs versus S-EVs ****P < .0001. (F) Histograms representing the percentage of responding cells. S-EVs number of total cell analyzed *n* = 445, responding cells *n* = 197 (40.33% ± 14.63); L-EVs cells analyzed *n* = 278, responding cells *n* = 195 (76.02% ± 16.51); L-EVs preincubated with AbCX43E2 *n* = 46, responding cell *n* = 8 (14.79% ± 8.54); total number of cells incubated with AbCX43E2 *n* = 31, number of responding cells *n* = 10 (32.26%); (G) Representative Western blot of PYK2 Y405 phosphorylation at basal levels or in response to L-EVs stimuli over time, *N* = 3. (H) Phospho-PYK2 (Y405) and (I) phospho-FAK (Y397) (see Supplementary Figure 4G) kinetic following L-EVs administration or without treatment. All time points are normalized on untreated cells at 5 min. Unpaired *t* test was applied to perform statistical analysis: L-EVs versus no treated: 5’ **P = .0090, 15’ ***P = .009, 30’ **P = .0069, 3 h **P = .0012. Graphs represent mean ± SE. (J) Relative cell number moving out of the neurospheres in ICH1 line after 24 h from a single L-EVs administration with or without PYK2 inhibitors (PYK2-IN-2 100 nM or PF-5622715 20 nM). L-EV *N* = 2 (*n* = 17), L-EV + PYK2-IN-2 *N* = 2 (*n* = 10), L-EV + PF-5622715 *N* = 2 (*n* = 6). Statistic was generated using Mann–Whitney test. L-EV versus L-EV + PYK2-IN-2 ***P = .0003; L-EV versus L-EV + PF-5622715 **P = .0099. Bars in graphs represent mean ± SE. (K) PCA analysis of MS/MS metabolomics data of S-EV and L-EV samples (S-EV *N* = 11, L-EV *N* = 7). (L) Differential enrichment of polar metabolites between L-EVs and S-EVs samples, as resulting from MS/MS metabolomics analysis, showing glutamine and glucose among others enriched molecules in L-EV. (M) Graphical abstract: large extracellular vesicles (L-EVs) trigger Cx43-dependent intracellular signaling and cell migration in glioblastoma cells. L-EVs increase the motility of recipient cells through an autocrine loop mediated by the formation of Cx43 gap junctions (GJ). Newly formed connexons enable the transfer of the L-EV cargo, enhancing calcium transients within recipient cells. The resulting calcium activation stimulates PYK2 phosphorylation on Y402 residue, directly promoting cell migration.

Glioblastoma cell migration and invasion are critically dependent on calcium signaling.^54^ We thus assessed whether calcium activity was involved in the L-EV-mediated enhancement of GBM cell migration. ICH1 cells, loaded with the dye Oregon Green, a light-excitable cell-permeant calcium indicator, were recorded in steady-state conditions and after EV treatment (Figure 6A). ICH1 displayed spontaneous Ca2^+^ transients which significantly increased in frequency upon exposure to 5 × 10^9^/mL of L-EVs (Figure 6C and E) but not upon exposure to the same amount of S-EVs (Figure 6B and E). Importantly, calcium transients were blocked by thapsigargin, a specific, irreversible inhibitor of endoplasmic reticulum Ca2^+^ ATPases (Figure 6B and C), indicating that L-EVs induced calcium dynamics resulted from intracellular calcium stores. Preincubation with AbCX43E2 significantly reduced L-EV ability to boost calcium waves (Figure 6D and E), further demonstrating the dependence of L-EVs-induced migration to the potentiation of calcium transient. The blocking antibody per se did not exhibit activity in modulating calcium dynamics (Figure 6E). Furthermore, L-EVs were able to increase calcium frequency in a higher percentage of target cells (~75%) with respect to S-EVs (~40%), with again no effect of the blocking antibody per se (Figure 6F).

Focal adhesion kinase (FAK) and its close paralogue, proline-rich tyrosine kinase 2 (PYK2), are key regulators of aggressive spreading and metastasis of cancer cells. Despite sharing overlapping cellular functions, PYK2 differs from FAK for its unique function of Ca2^+^ sensing. PYK2 acts as a central transducer of Ca2^+^ signals at cell contacts, where it induces focal adhesion disassembly.^55^ Auto-phosphorylation of the calcium-dependent kinase PYK2 stimulates glioma cell migration.^56^ Given that L-EVs promote calcium transients in GBM cells, PYK2 and FAK phosphorylation status was investigated at different time points after ICH1 cell exposure to L-EVs. Consistent with our hypothesis, we found a selective, time-dependent phosphorylation of PYK2 at residue Y402 upon challenge with L-EVs (Figure 6G and H). Conversely, FAK phosphorylation was not altered after L-EV exposure (Figure 6I and Supplementary Figure 4G). Furthermore, the specific PYK2 inhibitors PYK2-IN-2 (100 nM) or the PYK2/FAK inhibitor PF-562271 (20 nM) prevented the pro-migratory effect induced by L-EVs, further supporting PYK2 signaling activation downstream to L-EV exposure and calcium elevations (Figure 6J and Supplementary Figure 4I). Moreover, the percentage of cells displaying PYK2 phosphorylated upon challenge with L-EVs are reduced upon E2 blocking antibody (Supplementary Figure 4H).

To get some insights into the content of L-EVs and S-EVs, 4 different cell lines were processed for unsupervised untargeted identification at MS/MS. The most prominent polar metabolites potentially able to flux through Cx43-GJs were identified by generating MS/MS spectra. Results indicate a clear divergence in metabolites cargo between L-EVs and S-EVs, and a significant clustering of EV based on their subtype, irrespectively to patient origin (Figure 6K). The identified molecules were mostly ascribable to mediators involved in cell metabolic function, such as glucose and glutamine (Figure 6L and Supplementary Figure 4J). These findings acquire specific relevance as migration is an energetically demanding process requiring significant metabolic activity.^57^ Furthermore, glucose, contained in L-EVs but not S-EVs, is known to increase cytosolic Ca2^+^ levels.^58^

Together these data demonstrate that L-EVs drive GBM cell migration via Cx43-GJs operating on calcium signaling. Although further investigation is required, our data provide the proof-of-concept that L-EVs contain both calcium-elevating signaling molecules (Figure 6L) as well as the complete Cx43 machinery required for their transfer (Figure 5F and G).

## Discussion

It is acknowledged that the infiltrative nature of GBM limits therapeutic efficacy and promotes aggressive disease progression.^59^ Despite its therapeutic and prognostic significance, the process of GBM invasion is only partially understood and no effective strategy to prevent tumor cell invasive behavior has been unveiled. This encourages the study of GBM cell invasiveness as an utmost priority. In this study, we demonstrate both in vitro and in vivo that L-EVs released by GBM cells represent a relevant pro-migratory input for the tumor.

The role of GBM-EVs in modulating tumor behavior has been previously proposed to occur through the degradation and remodeling of the ECM,^20^ the reprogramming of cells residing in the tumor microenvironment,^60,61^ and/or the conditioning of a tumor-supportive niche.^62^ Furthermore, it has been shown that EVs generated by non-tumor cells in the microenvironment can exacerbate the migration ability of GBM cells.^63^ Although we cannot exclude the possible involvement of ECM remodeling, our study unveils a novel, self-sustaining, rapid mechanism exploited by GBM cells to trigger migration through autocrine release and engagement of a specific population of EVs, the L-EVs, through a process that involves the formation of Cx43-GJ mediated connections.

The L-EV-induced pro-migratory effect occurred in a time- and concentration-dependent manner and was reproduced using spheroids formed by cells derived from different patients. Furthermore, and in line with the growing body of literature indicating that different GBM molecular subtypes are characterized by distinct migratory capacity,^5,64^ we showed that cells with mesenchymal subtype, containing higher Cx43 expression compared to proneural cells, are particularly susceptible to the exposure of autologous L-EVs. Furthermore, by exploiting our human GLICOX assembloids, which offer a functional system to evaluate the integration of intrinsic cellular properties and external factors that can modulate GBM behavior,^65^ we provide a model to mimic the tumor-brain cell interactions. Furthermore, we confirmed that L-EVs promotes spreading of GBM cells also in a human 3D model.

A major contribution of our study is the clear step we made into the clinical setting through the isolation, characterization and functional assessment of EVs derived from the surgical fluids of patients. Cells from the PBZ are, in most of the cases, unresectable.^66^ The standard therapy is the surgical resection of the contrast-enhancing tumor component based on magnetic resonance imaging.^67,68^ More impactful is the supratotal resection, defined as extension of the resection to the surrounding FLAIR hyperintensity area.^4^ In this context, the use of a CUSA allows further tumor removal through ultrasonic tissue fragmentation. Large size-EVs, but not S-EVs isolated from the patient surgical aspirate, showed a strong pro-migratory effect. Our data strengthen the growing evidence indicating that the surgical aspirate represents a potential source for correlating circulating EV markers with histological, neuroradiological, and clinical parameters.^69–71^

Besides containing large amounts of EVs produced by tumor cells, the CUSA surgical aspirate contains cellular products originating from immune cells. Indeed, the immune system makes up a significant proportion—around 30%—of the total cells present in GBM.^72^ This prompted the need for characterizing the cellular origin of the L-EVs derived from patient surgical aspirates and promoting the tumor migration. Our approach exploited the expression of leukocyte antigen CD45 to isolate and separate CD45-positive and CD45-negative EVs present in surgical aspirates. Our data showing that CD45-negative L-EVs promote tumor migration in the spheroid assay, while CD45-positive L-EVs and S-EVs are ineffective, confers a high degree of specificity to the examined process.

It must be noted that CD45-negative L-EVs may originate, besides tumor cells, also by non-tumoral cell populations including endothelial cells and astrocytes. However, compared to non-cancerous cells, GBM cells release more EVs (approximately 10 000 EVs per single GBM cell over a 48-h period^73^). Moreover, tumor cells typically make up ~50–70% of the total cellular population in GBM,^74^ with the remaining proportion consisting prevalently of resident or infiltrated immune cells (30–50%),^75^ suggesting that L-EVs are prominent of tumoral origin. However, the possible additional processes contributed by L-EVs derived from the immune component of the tumor deserve further investigation.

Importantly, the boosting role of L-EVs on tumor invasiveness is detectable also in the in-vivo setting, where mice were treated with EVs through intranasal delivery, which allows them to distribute efficiently at tumor sites compared to the intravenous administration.^76^ Through this route, EVs can be absorbed by olfactory sensory cells via endocytosis, migrate via axonal transport, and are eventually released into the brain’s olfactory bulb. Alternatively, EVs might pass through the nasal epithelium’s tight junctions into the lamina propria, and then, moving externally along axons and enter directly into the CNS.^77^ After 8 weeks, we observed that mice treated with L-EVs showed tumors with higher invasiveness compared to the control group, with a more prominent number of cells detaching from the central lesion and invading the brain parenchyma.

In this study we show that the autocrine interaction between GBM cells and GBM-derived L-EVs relies on the formation of Cx43-GJ mediated connections, a mechanism previously described to mediate the GBM-to-GBM and the GBM-to-astrocyte cell-to-cell communication.^5,78^ Consistently, the Cx43 encoding gene is more prominently expressed in the mesenchymal molecular subtype, in line with their higher responsiveness to L-EV exposure. Although the vast majority of Cx43 is expressed at the membrane level in both the small and large EV fractions, only in L-EVs CX43 molecules appear to be assembled in functional hemichannels. Indeed, L-EVs contain a higher amount of Cx43 as detergent insoluble form, which is specifically associated with functional GJs.^46^ Also, L-EVs predominantly express PCDH7 that promotes the functional assembling of Cx43-mediated GJs.^47^ It is possible that the unstable membrane fraction of Cx43 and their consequent endocytosis in “connexosomes”^79^ may result in Cx43 degradation upon fusion with endosomes, followed by their entering into the pathways of multivesicular bodies.^80,81^ Despite the observations that small EVs do not express functional Cx43 hemichannels and do not trigger GBM cell motility, a role of S-EV in the GBM context cannot be excluded. Mechanisms subtending S-EV induced GBM growth and invasiveness independent from the transfer of signaling through Cx43-GJs have indeed been reported.^82^

In conclusion, we have unveiled an L-EV-based, autocrine mechanism which promotes tumor migration and invasiveness. Also, we have demonstrated its clinical relevance by showing the presence and functional efficacy of pro-migratory L-EVs of nonimmune origin in the patient surgery fluids. Finally, we provided molecular hints about the mechanisms involved. Although we cannot exclude additional mechanisms responsible for L-EV-induced migration enhancement, such as a role of membrane lipids, we propose the requirement of newly formed Cx43-GJs and the possible transfer of signaling molecules, which in turn increase intracellular calcium and cell migration (Figure 6M). Our data provide different molecular targets suitable for attempting a limitation of tumor invasiveness, which is particularly relevant in the peripheral area of the tumor, where recurrence mostly originates from.

## Supplementary material

Supplementary material is available online at *Neuro-Oncology* (https://academic.oup.com/neuro-oncology).

noaf013_Supplementary_Mathods

noaf013_Supplementary_Figures

## Data Availability

All data associated with this study are presented in the manuscript or in the Supplementary Materials.
