# Supplementary material for: Extracellular vesicles released by glioblastoma cancer cells drive tumor invasiveness via Connexin-43 gap junctions
Source: Neuro Oncol. 2025 Jan 30;27(11):2843–60. doi: 10.1093/neuonc/noaf013 (PMC12908494; doi:10.1093/neuonc/noaf013)
Supplement: noaf013_Supplementary_Mathods [file noaf013_supplementary_mathods.docx]

**Supplementary Figures**

**Supplementary Fig. 1 Characterization of EVs derived from GBM cells and their involvement on cell spreading and motility. (A)** Mesenchymal index after Z-score correction of the GBM cell lines involved in the present study. **(B)** Size distribution of ICH1 EVs obtained by electron microscopy analysis. **(C)** Average diameter of neurospheres measured after 24h of colture in GFs depleted medium. ICH1 n=36, ICH2 n=23, ICH3 n=32. (D) Visual description of the ROI used to score migrating cells highlighted in red: (right upper panel) ROI1 the whole field. (right lower panel) ROI2 the outer region, a variable ROI. **(E)** Effect of growth factors depletion on cell proliferation rate. MTT assay to detect cell growth (N=3, n=6), clonogenic assay (n=5). Scale bar of 100 µm. **(F)** Spheroid migration assay. Histograms indicate the mean ± SE of cells out of the ICH2 and ICH3 neurospheres after 24 hours from plating following a single dose of EVs treatment. ICH2 Not treated N=2 (n=23), S-EVs N=1 (n=17), L-EVs N=2 (n=45). ICH3 Not treated N=4 (n=32), S-EVs N=2 (n=46), L-EVs N=4 (n=83). Two-way ANOVA Bonferroni’s multiple comparison test was used for statistical analysis on cumulative data: ICH2 L-EVs vs s-EVs 5x10^9 p<0.0001****, L-EVs 5x10^9 vs Not treated p<0.0001****. ICH3 L-EVs vs S-EVs 5x10^9 p=0.0010***, L-EVs 5x10^9 vs Not treated p<0.0001****. **(G)** Cells count 48 hours after EVs treatment performed on neurospheres treated with 1x10^9 EVs. ICH2 not treated N=2 (n=22), S-EVs N=1 (n=4), L-EVs N=1 (n=5). ICH3 Not treated N=3 (n=18), S-EVs N=2 (n=10), L-EVs N=3 (n=15).; One-way ANOVA with Tukey correction test was used for statistical analysis; ICH2 L-EVs vs Not treated p<0.0001****, L-EVS vs S-EVs p<0.0001****; ICH3 L-EVs vs Not treated p<0.0001****, L-EVS vs S-EVs p=0.0022**. Histograms represent the mean number of cells ± SE.

**Supplementary Fig. 2 Generation and characterization of GLICOX assembloids. (A)** Schematic of the experimental setup displaying GBM spheroid ICH1-GFP+ and cortical organoids (hCOs) assembled together (GLICOX). Scale bar 500µm. **(B)** Schematic of the IMARIS pipeline for quantification of spheroid area and volume. After ROI selection (GBM spheroid) a surface mask was applied to extract area and volume parameters for statistical analysis. **(C)** Representative images of GLICOX showing the whole organoid (dotted line) and the GBM spheroids at different time points (24, 72 and 96 hrs). Scale bar, 250 µm. IMARIS quantification of GBM spheroid area at the different time points shows a significant increase of area at 96hrs (4 to 6 GBM spheroids quantified). **(D)** GLICOX sectioning shows the invasion of ICH1 GFP+ cells into the organoid, representative examples are reported. Scale bar, 250 µm. Schematic of the dynamics of GBM invasion in cortical organoid. **(E)** Schematic of the experimental setup displaying the treatment of GLICOX with L-EVs from the start of the culture to the endpoint (96hrs). **(F)** Analysis of RFP-Labeled L-EVs after 24 hours of treatment: confocal images showing no signal in Not treated condition and reduced pattern of RFP-labeled L-EVs in a field depicting no GFP-positive GBM cells. **(G)** Representative micrograph relative to panel Figure 1K.

**Supplementary Fig. 3 Characterization of EVs derived from ICH27 and CUSA aspirate following CD45-depletion strategy and MACSPlex approach. (A)** NTA analysis of the size distribution and quantity of EVs spontaneously released by patient-derived GBM cell ICH27-PBZ. Graphs refer to the estimate of EVs isolated from 1x10^6 cells in 24 hours in growth factors depleted medium (N=3). **(B)** Representative western blot of EVs markers expression on ICH27-PBZ. Blot shows enrichment of biogenetic markers in small and large vesicles. **(C)** Fold change of the mean cells out of the sphere at 24h from L-EV treatment compare to Not treated condition. Cell lines were stratified on the basis of their transcriptional subtype. Unpaired t-test; p=0.0544. **(D)** NTA analysis of the size distribution and quantity of EVs isolated from **patient 27** surgical aspirate (10 ml; N=3). **(E)** Concentrations of S-EVs and L-EVs isolated from surgical aspirates obtained from six different patients, based on a 10 mL sample volume. (F) Heatmap visualization of markers RFI detected through MACSplex analysis on extended sample collection of surgical aspirate derived from different patients. **(G)** Capture bead distribution, gating strategy and APC-MFI shift after 1 hour of incubation with EVs from surgical aspirate. **(H)** NTA analysis of the size distribution and quantity of EVs isolated from patient 27 surgical aspirate following the CD45-depletion (N=3).

**Supplementary Fig. 4 GJ-dependent L-EVs blocking strategies and GJ characterization. (A)** Spheroid migration assay quantification of the pro migratory effect of CBX (6 µM) on neurospheres. Not treated N=1 (n=8), CBX N=1 (n=6). **(B)** Representative Cx43 immunoblot in small and large EV population derived from ICH2 (N=5) and ICH3 (N=4) cells. **(C)** Representative immunoblots showing PCDH7 on ICH2 (N=4) and ICH3 (N=5) derived EVs. Integrated density of the bands is normalized on stain free signal; histograms show mean± SE; **(D)** GBM cell state hierarchy plot as defined by Neftel ^1^ displaying *GJA1* or *CD44* expression across diverse states. Oligodendrocyte progenitor-like (OPC-like), neural progenitor like (NPC-like), astrocyte-like (AC-like), and mesenchymal-like (MES-like). **(E)** Positive expression correlation between *GJA1* and *CD44* transcript according to TCGA-GBM publically available dataset. Spearman correlation *p=0.02. **(F)** Freeze thaw experiments performed on L-EVs isolated from ICH1. Upper panel: NTA analysis of the size distribution and quantity of input and post freeze and thaw cycles EVs, with specification of median vesicles dimensional sizes. Bottom left panel: migration test performed using L-EV or L-EV-derived membrane fraction obtained by freeze thaw protocol. N=1; not treated n=6. Treated with L-EV n=6, treated with L-EV membrane fraction n=6. Unpaired T test: whole L-EV vs not treated **p=0.0025; whole L-EV vs L-EV-membrane **p=0.0092; L-EV-membrane vs not treated ns p=0.132. Bottom right panel: Western blot showing CX43 detection on L-EVs lysate and into membrane fraction disrupted by freeze thaw technique. **(G)** Representative western blot of FAK Y397 phosphorylation at basal level or following L-EVs treatment. All time points are normalized on untreated cells at 5 minutes. N=3. Unpaired T test was applied to perform statistical analysis. All error bars in graphs represent mean±SE. **(H)** Percentage of positive cells according to phopsho-PYK2 staining at flow cytometry in not treated, L-EVs, L-EV+blocking antibody AbCxE2 and L-EV+isotype control antybody (N=1). **(I)** Relative cell number moving out of the spheres after 24h without or with PYK2 inhibitors (PYK2-IN-2 100 nM or PF-5622715 20 nM). Not treated N=2 (n=14), PYK2-IN-2 N=2 (n=13), PF-5622715 N=2 (n=14). Statistic was generated using Mann-Whitney test. **(J)** Normalized concentration of Glutamine and Glucose in S-EV and L-EV samples according to MS/MS quantitation (S-EV N=11, L-EV N=7).

**SUPPLEMENTARY MATERIAL AND METHODS**

**Patient-derived GBM cell lines and subtype characterization**

Isolated cells were cultured in Neurocult Proliferation medium (Stem Cell) supplemented with hormone mix (Stem Cell), 20 ng/ml Human basal FGF, and 20 ng/ml EGF (Peprotech) and were seeded in flasks coated with poly(2-hydroxyethyl methacrylate) (Sigma). All established lines were used within passage 15 and tested negative for mycoplasma infection (Euroclone, N-GARDE Mycoplasma PCR Reagent set).

Cell line molecular subtyping was performed by TaqMan® gene expression assays (Applied Biosystems™, ThermoFisher Scientific) using manually curated gene sets defining proneural (PN) and mesenchymal (MES) state in GBM ^2^. Briefly, a set of four PN (*SOX2*, *ADGRG1*, *PROM1*, and *OLIG2*) and five MES genes (*CHI3L1*, *CTGF*, *FN1*, *CD44* and *CD109*), were considered. *GAPDH* was used as housekeeping gene. Gene expression was then z-score corrected and mesenchymal index calculated ^3^.

**EVs isolation and characterization**

Cells were removed from medium at 300 g at room temperature (RT) for 5 minutes, apoptotic bodies and debris were cleared following centrifugation at 1,200 g at 4 °C for 20 minutes. L-EVs were pelleted at 13,000 g for 30 minutes at 4°C. S-EV were then isolated from the remaining supernatant through serial ultracentrifugation in polypropylene tubes (Beckmann) at 110,000 g for 70 minutes at 4°C (Beckman Optima L-90K Ultracentrifuge equipped with SW 40 Ti Swinging Bucket Rotor or Thermo Scientific Sorvall WX+ Ultra Series equipped with TH-641 Swinging Bucket Rotor). EVs from surgical aspirates were processed likewise. EV fresh preparations were used for cellular functional experiments (i.e. migration assay, calcium imaging, PYK-2/FAK phosphorylation), while EVs were snap-frozen for biochemical assessments.EV size distribution was evaluated by Nanoparticles Tracking Analysis (NTA) and electron microscopy. NTA was performed using Nanosight NS300 (Malvern Panalytical Ltd) equipped with 488 and 532 lasers. The acquisition threshold level was set in accordance to supplier guidelines in order to have a mix of saturated and not saturated particles. Five Videos of 60 seconds were collected setting the 1 ml syringe pump speed to 30 units.

For electron microscopy negative staining ^4^, EV preparations were resuspended in 20 μl PBS (pH 7.4) and fixed by adding an equal volume of 2% paraformaldehyde in 0.1 mol/l phosphate buffer (pH 7.4). EVs were then adsorbed for 10 minutes to formvar-carbon coated copper grids by floating the grids on 5 μl drops on parafilm. Subsequently, grids with adhered vesicles were rinsed in PBS and negatively stained with 2% uranyl acetate for 5 minutes at room temperature. Stained grids were embedded in 2.5% methylcellulose for improved preservation and air-dried before examination. Electron micrographs were taken at Hitachi TEM microscope (HT7800 series, Tokyo, Japan) equipped with Megaview 3 digital camera and Radius 2.0 software (EMSIS, Germany). Morphometry analysis of the size of EVs was measured on 15 randomly taken micrographs for each condition and calculated using the arbitrary line function embedded in the measurement dialog box of Radius 2.0 software (EMSIS, Germany)^5^. To visualize EV size distribution, the results were plotted as a frequency distribution histogram and as a scatter dot plot in which each size measured is represented as a point along with lines for the median value and the range.

**Spheroid migration assay acquisition and analysis**

Spheroids pictures were taken at 4x magnification 24 and 48 hours after seeding with CellR Widefield Microscope (Olympus) or EVOS Cell Imaging Systems (Thermo Fisher Scientific). Total number of cells out of the spheres was counted on two different regions of interest (ROI): 1) ROI1 - the whole field of view coinciding to a fixed rectangular area of 3.58 mm^2^ (Fig. 1F - upper panel, area highlighted in red); 2) ROI2 - a variable field encompassing the farthest area below a distance equal to each neurosphere diameter (Fig. 1F - lower panel, area highlighted in red). Cell density in the ROIs was counted and recorded using Image J software (plugin cell counter).

**Proliferation detection**

To detect proliferation of GBM cell lines under the same condition of EVs stimulation a gliomaphere formation assay and an MTT assay were performed as follow. Cells were seeded as single-cell suspension at clonal densities 0.5 cell/µL. Plates were visually scanned after 14 days under light microscope and neurosphere size recorded. For residual metabolic activity cells were seeded at 50 cells/ul density and MTT conversion was measured after 7 days at a final concentration of 50 ug/ml (SIGMA).

**Western blotting**

Cells and EVs were lysed in RIPA buffer (1% triton x 100, 50 mM Tris pH 7.4, 1 mM EDTA pH 8.8, 500 mM NaCl) supplemented with protease and phosphatase inhibitors (Roche). Staining of EVs markers Alix (Cell Signaling Technology, cat. 2171S), TSG101 (Millipore, cat. T5826), Actin (Santa Cruz Biotechnology, sc-1615), CD9 (Cell Signaling Technology, cat. 131745), CX43 (Merck, cat. C6219) and protocadherin 7 (PCDH7) (Abcam, ab139274) detection was carried out in reducing conditions. Meanwhile, CD63 (Santa Cruz Biotechnology, sc-5275) and Integrin B1 (Sigma, SAB4300655) detection were carried out in non-reducing conditions. Quantification of protocadherin 7 (PCDH7) (Abcam, ab139274) was obtained by normalizing chemiluminescent signal on stain-free. Lysis buffer (50 mM tris pH 7.4, 1 mM EDTA pH 8.8, 500 mM NaCl) depleted of detergents was used to separate (30 minutes at 20.000 g) the membrane fraction of Cx43, in the pellet, from the intraluminal fraction one in the supernatant. The pellet was resuspended in 10 ul of an equal mixture of RIPA 2x and SDS 10% RT. The supernatant was diluted in 5 ul of H_2_0 and triton x-100 2% to obtain RIPA 1x buffer. To separate Cx43 soluble and insoluble forms the protocol reported by Manta Rei et. al ^6^ was modified as follows. EV lysates (RIPA buffer) were centrifuged for 30 minutes at 20,000 g 4°C to separate soluble fraction (supernatant) and triton-x 100 insoluble fraction (pellet). The pellet was washed once with cold PBS 1X or RIPA buffer and centrifuged again (30 minutes to 20,000 g 4°C) and then resuspended in a mixture of RIPA buffer 2x and SDS 10% in equal proportion.

Evaluation of Focal adhesion complex was performed plating in high density cell culture 400,000 cells/well (48-well plate) coated with fibronectin (5 ug/ml) and collagen I (1 ug/ml) in medium w/o GFs. Activation of focal adhesion proteins was performed through detection of cytoplasmic (DOI: 10.1371/journal.pone.0025545) phospho-PYK2 (Tyr402) (Millipore, cat. 07-892) and phospho-FAK (Tyr397, cat. 700255) (Invitrogen). Signal intensities of phosphoproteins were normalized on their total protein, total PYK2 (Invitrogen, cat. MA5-15407) and total FAK (Invitrogen, cat. 39-6500) respectively.

Protein quantification was carried out using micro BCA (Thermofisher). 5 or 10 ug of proteins were charged on stain free precast gel (gradient 4-20%) or 10-12% stain free gels (Biorad) and separated with electrophoresis. Proteins were then blotted (fast blot Biorad) on nitrocellulose membrane. Signal development was carried out by chemiluminescence ECL clarity (Biorad) or ECL femto (thermofisher) according to the manufacturer’s instructions. Chemiluminescence was detected by a chemidoc system (Biorad) and the intensity of the signal was quantified by Image Lab software (Biorad). Representative western blot images were lightened with Fiji software (NIH, Bethesda, Maryland, USA; https://imagej.net/learn/brightness-and-contrast).

**Generation of 3D human cortical organoids**

Cortical organoids were generated as described by Velasco and colleagues^7^ with minor modifications. In brief, hES cells were cultured and checked for stem cell colony morphology and normal karyotype. At around 90-80% confluency, cells were enzymatically dissociated with Accutase (Gibco) into single cells, and 9,000 cells per organoid were grown in a 96-well microplate with v-bottom wells (Amsbio) in cortical differentiation media I (CDMI) containing Glasgow-MEM (Gibco), 20% Knockout Serum Replacement (KSR) (Gibco), 0.1 mM Minimum Essential Medium non-essential amino acids (MEM-NEAA) (Gibco), 1 mM pyruvate (Gibco), 0.1 mM 2-mercaptoethanol (Gibco), 100 U/ml penicillin (Corning) and 100 µg/ml streptomycin (Gibco). From day 0 to day 6 CDMI was supplemented with 20μM ROCK inhibitor (Aurogene), 3μM of WNT inhibitor IWR1 (Calbiochem), and 5μM of TGFβ inhibitor SB 431542 (Stemcell Technologies), while from day 6 to day 18 CDMI was supplemented only with 3μM IWR1 and 5μM SB 431542. At day18 cortical organoids were transferred into ultra-low attachment dish and grown under orbital shaking at 70rpm in CDMII, containing DMEM/F12 medium (Gibco), 2 mM Glutamax (Gibco), 1% N2 (Gibco), 1% Chemically Defined Lipid Concentrate (Gibco), 0.25 µg/ml fungizone (Gibco), 100 U/ml penicillin and 100 µg/ml streptomycin. On day 35, organoids were grown in CDM III, consisting of CDM II supplemented with 10% fetal bovine serum (FBS) (Gibco), 5 µg/ml heparin (Sigma) and 1% Matrigel (Corning). From day 70, organoids were cultured in CDM IV, consisting of CDM III supplemented with B27 supplement (Gibco) and 2% Matrigel. Aggregate formations, growth and cortical differentiation was checked at critical time points.

**Generation of GLIOCOX assembloids**

GBM neurospheres were generated by seeding 800 to 2,000 glioblastoma cells in low adherent ‘‘U’’ shaped microwells in CDMIII without the addition of FBS (Glicox-CDM). GBM neurospheres were allowed to aggregate for 48-96 hrs before fusion with cortical organoids.

2-months cortical organoids produced as indicated in supplementary materials were fused with ICH01-GFP neurospheres, two neurospheres composed of 800 cells and one of 2,000 cells. Assembled GLICOX were treated for 96 hrs with vesicles (5x10^9^ L-EVs/ ml or 5x10^9^ S-EV/ml) while controls were maintained in GLICOX-CDM to compare the invasion capacity of ICH1 upon treatment. 4/5-months cortical organoids were fused with one ICH27-TC (RFP+) and ICH27-PBZ (GFP+) neurosphere composed of 800 cells. Assembled GLICOX were monitored for 30 days to assess the invasive capacity of these two glioblastoma populations in a 3D microenvironment with similarities to the human tumor microenvironment.

2-months cortical organoids produced were fused with 1 ICH01-GFP neurospheres of 2,000 cells as described before. Assembled GLICOX were treated for 24 hrs with microvesicles derived from ICH01- RFP (10*109 L-EVs/ ml).

**GLICOX Live imaging**

Images of organoids in culture were taken on an EVOS FL microscope (Invitrogen). For organoid live imaging at different daytime points, confocal microscope laser inverted scanning Leica SP8II was used. Images were processed with QuPath 0.4.4 and Fiji, where maximum intensity projections were generated. IMARIS software (Oxford instruments, v 9.7.2) software was used for the quantification of the area and volume of the spheroids over time.

**Lentiviral Particle Generation and cells Transduction**

Lentiviral particles were generated by co-transfection into HEK293T of plasmids pRRL. CMV.Luciferase.ires.eGFP (kindly provided by De Bacco’s laboratory, Laboratory of Cancer Stem Cell Research, Candiolo Cancer Institute, FPO-IRCCS, Turin, Italy) or pLV[Exp]-CMV>Luciferase:IRES:mRFP1 (Vectorbuilder) with lentiviral packaging vectors (ViraPower™ Lentiviral Expression Systems, Invitrogen) using the Lipofectamine 2000 (Invitrogen) in OPTIMEM reduced serum medium (GIBCO). Cell culture supernatant, once collected, was filtered (0.45 µm) and purified by centrifugation at 50000× g for 2 h at 4 °C. Particles were resuspended in PBS and cells infected for 1 h at 37 °C. Infected cells were selected by means of GFP-pos selection at BD FACSMelody™ Cell Sorter (BD Biosciences), or RFP-pos selection at BD FACSAria III Cell Sorter (BD Biosciences).

**Culturing and maintenance of human ES and hiPSCs.**

Human embryonic stem cells (hES) line RUESe002-A (Rockefeller University Embryonic Stem cell line 2) and human induced pluripotent stem cells (hiPSC) line GM23338*D (Coriell Institute) were used in this study to generate 3D human cortical organoids. hES and hiPSC lines were plated on Geltrex or Vitronectin, respectively (Gibco), coated cell culture dishes for 30 min at 37°C and 5% CO2 using Essential8 or Stemflex medium (Gibco). Cells were passed with Gentle Cell Dissociation Reagent (Stemcell Technologies), mechanically dissociated into small aggregates and re-cultured in 1:6 dilution into fresh coated dishes every 3-4 days.

**Enrichment/depletion of CD45-positive EVs**

After isolation from surgical aspirate, EVs were incubated for 1h at room temperature with CD45 microbeads in the optimized ratio of 1:1.5 (EV:microbeads). After incubation, the EVs were magnetic separated placing the µColumns on the µMACS Separator (Miltenyi) held by the MACS MultiStand (Miltenyi) and equilibrated with 100µl of equilibration buffer. After equilibration, magnetically labelled sample run through onto the column and negative fraction of EVs for CD45 negative were recovered. Finally, the columns were removed from the MACS MultiStand and the columns placed on 1.5 ml Eppendorf. Without magnetic field generated by µMACS Separator the microbeads into the columns were eluted with 100µl of isolation buffer and EVs CD45 positive.

**Assessment of multiplex Beads-Based flow cytometry assay (MACSPlex)**

EV fractions from surgical aspirate were subjected to bead-based multiplex analysis by flow cytometry (MACSPlex EVs Kit, human, Miltenyi Biotec - 130-108-813). EV-containing samples were processed following manufacturer instruction according to the short protocol for 1.5 mL reagent tubes. Samples were diluted with MACSPlex buffer to a final volume of 120 μl. Next, 15 μl of MACSPlex EVs Capture Beads (containing 37 different antibody-coated bead subsets) were added to each sample and incubated for 1h at room temperature rotating at 12 rpm protected from light. For counterstaining of EVs bound by capture beads with detection antibodies, 135 μl of MPB and 5 μl of each APC-conjugated anti-CD9, anti-CD63, and anti-CD81 detection antibody were added to each sample and plates were incubated rotating at 12 rpm protected from light for 1 h at room temperature. After several washing steps, according to the manufacturer's protocol, the samples were resuspended by pipetting up and down and transferred to the FACS tube for analysis. Two biological replicates were performed for each sample. Flow cytometry analysis was performed with an LSR Fortessa cytometer (BD Biosciences). FlowJo software (v10, FlowJo LLC) was used to analyze flow cytometry data collecting at least 10,000 events. Median fluorescence intensity (MFI) for all 37 capture beads were background corrected by subtracting respective MFI values from matched non-EV negative controls, thus obtaining relative fluorescence intensity (RFI) values.

Heatmap visualization of MACSplex RFI values was generated using pheatmap v1.0.12 R package (hierarchical clustering is based on Euclidean distance and values were not scaled).

**Cell circularity quantification**

Quantification of cells circularity, was performed at DIV7 by image analyses carried out by ImageJ software (1.51i Version) follow those steps: i) Process 🡪 Substract Background; ii) Image 🡪 Adjust 🡪 Threshold; iii) Process 🡪 Binary 🡪 Watershed; iv) Analyze 🡪 Analyze Particle.

**Brain tissue and GLICOX processing and immunostaining evaluation**

Brains were fixed overnight in 4% paraformaldehyde at 4°C and subsequently transferred to 30% sucrose/PBS for cryoprotection. GLIOCOX samples were fixed in 4% paraformaldehyde (DBA) plus 4% sucrose for 10 minutes and cryoprotected by performing a scale of 10%, 20% and 30% sucrose solutions, embedded in optimum cutting temperature (OCT) compound (Histo-Line Laboratories). Primary antibodies used: GFP 1/1000 (Aves Labs, GFP-1010; 1:400, Invitrogen, cat. A6455) and RFP 1/500 (Rockland, 600-401-379S) and and mouse anti-human nuclei (1:200, Merck, MAB1261) primary antibodies. Sections were then incubated with secondary antibody solution (Alexa 488 goat anti-rabbit IgG (H+L); Alexa 568 goat anti-mouse IgG (H+L)) all used at 1:500 (Thermo-Fisher). Slices were stained with Hoechst (Thermo-Fisher, cat.62249). Brain and organoids pictures were taken using Thunder Imaging System (Leica-Microsystems) equipped with Leica-DFC9000GT-VSC09049 camera and HC PL FLUOTAR L 20x/0.40 or 0.80 dry objective. Fluorophores were excited with led wavelength excitation at 390, 475, and 555. A mosaic of whole coronal brain sections was taken by mosaic acquisition (minimum overlapping 25%). Immunofluorescent images of GLICOX sections were acquired using the automated microscope (Zeiss Axioscan Z1) using a 20X objective. To acquire GLICOX assembloids in 3D treated with RFP-positive microvesicles and control SP8 STED3X SMD (Leica) was used in confocal modality. The detection of tumoral central lesion, migrating cells, tumor margin and cellularity on mice brain sections was performed using Fiji software (NIH, Bethesda, Maryland, USA). To partially subtract background, the rolling ball background subtraction was used (https://imagej.net/Rolling_Ball_Background_Subtraction).

To obtain the margins of central lesion for each section (ROI, region of interest), GFP channels were binarized. Obtained ROIs were measured and roundness parameter quantified as described by means of circularity parameter adjusted by CAR ^8^.

$$R=circularity+(1-C_{AR})$$

$$C_{AR}=\frac{2*D*d}{D^{2}+d^{2}}$$

$$D= Major, primary axis of the best fitting ellipse$$

$$d= Minor, secondary axis of the best fitting ellipse$$

Tumor cellularity was measured within the main lesion using the boolean function “AND” of the image calculator tool between GFP and DAPI binarized channels. Nuclei were counted automatically using the analyze particle function.

Recognition of migrating cells out of the central region was performed through colocalization between GFP and Hoechst binarized signals using the AND function.

GLICOX images were processed with QuPath 0.4.4 and Fiji, section binning was performed in QuPath by a semi-automated process, and cell counting was performed manually for individual bins. Invasive protrusions were manually counted using QuPath.

Final images were processed using Adobe Photoshop CS5 extended (v12.1), and Adobe Illustrator (Adobe 2024).

**Inhibition of GJs in EVs**

EVs were incubated with 100 uM of Carbenoxolone (CBX) during 3 hours at 37°C. Treated EVs were then supplemented to spheroid (CBX final concentration 3.7 uM). The use of the Connexin-43 blocking antibody AbCX43E2 was previously described ^9^. EVs were pre-incubated with different antibody concentrations (10, 50 or 100 ug/ml) during 3 hours at 37°C. Incubated EVs were then diluted in neurosphere medium reaching a final concentration of EVs 5x10^9 /ml and blocking antibody resulted in a final concentration range of 0.07 to 7,1 ug/ml depending on the starting EVs concentration. Controls samples were treated with equal final concentration of blocking antibody. For calcium experiments L-EVs was pre-incubated with blocking antibody at the concentration of 50 or 100 ug/ml for three hours at 37°C. The final concentration of L-EVs/AbCX43E2 in KRH with cells in dose range from 0.5 to 3.5 ug/ml.

**Inhibition of PYK2 activity**

In order to prevents PYK2 activation were tested two different inhibitors: PyK2-IN-2 100 nM (MedChemExpress, Cat. HY-401485) or PF-562271 20 nM (MedChemExpress, Cat. HY-10459). Neurospheres were treated with inhibitors alone or at the same time with L-EVs and spheroids visualized after 24h for scoring of migrating cells.

**Nanogold immunolabeling of cells and EVS**

Cells and EVs were fixed in 4% paraformaldehyde (PFA) in phosphate-buffered saline (PBS) at room temperature. After fixation, cells were rinsed in PBS solution supplemented with 0.1% saponin (SAP) and 0.1% bovine serum albumin (BSA).Then, cells were incubated for 30 minutes in a blockin solution (BS) solution (0.5% BSA, 50 mM ammonium chloride (NH₄Cl), 0.1% saponin, 0.1% acetylated BSA (acBSA), and 150 mM NaCl in PBS). Following blocking, cells were incubated with anti-connexin 43 antibodies (1:200) (Merck, cat. C6219) diluted in blocking solution (BS) for 1 hour at room temperature. Following wash with BS, samples were incubated for one hour with a secondary antibody (1:100) conjugated to 1.4 nm NanoGold-Fab anti-mouse (aM) in BS (Nanoprobes, Inc. 95 Horseblock Road, Yaphank, NY, USA). To stabilize The gold labeling was stabilized in 2.5% glutaraldehyde (GA) for 15–20 minutes at room temperature, followed by a 10-minute wash in 0.5% BSA and 150 mM NaCl in distilled water (dH₂O). Nanogold particles were enlarged by sequential incubation in a gold enhancement solution for 10 minutes following the manufacturer’s protocol (Nanoprobes, Inc. 95 Horseblock Road, Yaphank, NY, USA). Cells were then incubated in 1% osmium tetroxide (OsO₄) in dH₂O for 1 hour at room temperature, rinsed with dH₂O, and stained with 1% uranyl acetate (UA) in dH₂O for 30 minutes. For dehydration, samples were gradually treated with 70% ethanol overnight, then three 5-minute washes in 95% ethanol, and three 5-minute washes in 100% ethanol. Finally, the samples were infiltrated with epoxy resin in three 1-hour steps, embedded, and polymerized at 60°C for 2 days. The protocol of EVs immunolabeling provides differents settings. Five microliter of concentrated EVs solution was dropped on formwar-coated copper grids and incubated for 20 min at room temperature, wash in PBS. In order to saturate the nonspecific reactives sites were performed two passages in 0.2% glycine in PBS for five minutes each. Then grids was blocked with 1% BSA in PBS for 5 min followed by incubation with the Anti-connexin43 antibody (1:200) (Merck, cat. C6219) for 30 min at room temperature. After four washes in 1% BSA in PBS of 2 min each, grids were incubated with 10-nm protein A gold (PAG) (Utrecht University, Cell Microscopy Core, The Netherlands) for 20 min. Then grids were washed four times in a solution of 1% BSA of two minutes each and then postfixed in 1% glutaraldehyde for 5 min followed by washes in PBS and in distilled water for 5 min.The samples were counterstained with 2% uranyl acetate in 0.15-M oxalic acid for 5 min at room temperature. The procedure was completed with a passage in 2.5% methylcellulose in uranyl acetate 4% for 5 min at room temperature, which allows to confer further contrast and to form a beam protective layer on the grids.

**Flow-cytometry analysis**

Cells were stained with conjugated rat antibody anti-CD44 (CD44-BV510 clone IM7, cat. 103043, Biolegend; 1:100) using BV buffer (cat. 566349, BD Biosciences) and then cells were subjected to intracellular staining following manufacturer instruction with the BD Cytofix/Cytoperm fixation and permeabilization kit (cat. 554714, BD Biosciences). Cells were then incubated with rabbit anti-Cx43 antibody (C6219; dilution 1:100, final concentration: 6 ug/ml, Sigma) or comparable concentration of rabbit IgG isotype control (cat. 02-6102, Invitrogen; 6 ug/ml). Unconjugated anti-Cx43 antibody was detected using goat anti-rabbit IgG Highly Cross-Adsorbed Alexa Fluor 633 (cat. A21071, ThermoFisher scientific; dilution 1:400). Flow cytometry analysis were performed with an LSR Fortessa cytometer (BD Biosciences). Dead cells recognition and removal from flow-citometry analysis was ensured with Zombie NIR™ Fixable Viability Kit (cat. 423105, Biolegend). FlowJo software (v10, FlowJo LLC) was used to analyze flow cytometry data collecting at least 10,000 events. Median fluorescence intensity (MFI) was background corrected by subtracting MFI values from isotype controls, thus obtaining relative fluorescence intensity (RFI) values.

**Freeze thaw**

L-EVs were subjected to 10 subsequent cycles of low freeze thawing in PBS 10x in order to maximize vesicles disruption. In order to collect membrane fraction, L-EV were then centrifuge to 20.000 g for 35 minutes 4°C. Membrane pellet was resuspended in PBS 1X.

**Calcium Imaging**

Calcium imaging was performed on cells loaded with Oregon Green 488 BAPTA (Thermo Fisher) as previously described ^10^. Briefly, cells were seeded on fibronectin and collagen I coated glass dishes and let to settle during 3 hours in Neurocult medium depleted of GFs. Subsequently cells were incubated for 1 hour with calcium sensitive dye (160 nM) and then glass loaded onto a recording chamber (Slotted Bath Chamber, RC-47FSLP, Warner Instruments). Recordings were performed in KRH on IX-71 inverted microscope (Olympus) equipped with 10x objective and an EMCCD (electron-multiplying CCD) camera (Quantem 512x512, Photometrics). Images of cells were taken at 480 nm excitation wavelengths. Recording of calcium waves were performed in basal condition, after administration of EVs and after treatment with 2 or 4 uM Thapsigargin (Sigma) in 200 ul of KRH in the holder. Time-lapse recording of each condition was obtained with an acquisition rate of 2 Hz for 300 s (binning = 2). Recording and analysis were performed using software MetaFluor software (Molecular Devices) (version 7.7.11.0). The traces were manually inspected and processed using custom Python scripts. Relative fluorescence (ΔF/F0) was computed as (Fc−F0)/F0. The baseline fluorescence (F0) was calculated as the mean of the fluorescence intensities observed across the initial five frames of the recording sequence. Baseline correction has been applied to compensate for photobleaching (polynomial fitting). The peak detection algorithm autonomously establishes the threshold at four standard deviations above the baseline noise level, dynamically adapts to the inherent variability present within the recording. Only tracks with stable Calcium waves and responding to Thapsigargin stimulus were considered in statistical analysis.

**Metabolomics analysis**

EVS pellet samples were extracted using 500 µL of acetonitrile/water (95:5, v/v) containing 50 mM ammonium acetate and 1 µM medronic acid at pH 10 with ammonium hydroxide. The mixture was vortexed and centrifuged at 15,000 g for 10 minutes at 4 °C. 250 µL of supernatant was collected and dried under nitrogen. The remaining pellet, containing denatured proteins, was dissolved in 8 M urea buffer, and the protein content was measured using the Pierce BCA protein assay kit (Thermo Scientific). The dried samples were reconstituted in 30 µL of 70:30 (v/v) ACN/H₂O at pH 10 with ammonium hydroxide prior analysis. UHPLC-MS Analysis was performed according to the following protocol. The reconstituted samples were analyzed using a UHPLC Vanquish system (Thermo Scientific) coupled with an Orbitrap Exploris 120 (Thermo Scientific). The LC-MS settings: column: Poroshell 120 HILIC-Z (2.1 x 100 mm, 2.7 µm, Agilent), 30°C, flow rate, Flow Rate: 0.200 mL/min,Mobile Phase A: Acetonitrile/water (5:95, v/v) with 50 mM ammonium acetate, 1 µM medronic acid, pH 10, Mobile Phase B: Acetonitrile/water (95:5, v/v) with 1 µM medronic acid, pH 10. Gradient (0–0.5 min: 70% B, 0.5–5 min: 70% to 5% B, 5–8 min: 5% B, 8–9 min: 5% to 70% B, 9–15 min: 70% B. Total Run Time: 15 min, ESI Mode: Negative, Source Voltage: 2.6 V, Capillary Temperature: 320 °C, S-Lens RF Level: 70. Data Acquisition: Data-dependent (ddMS2) top 4 scan mode, full-Scan MS Range: m/z 70 to 900, Resolution: 60,000 (full-scan), 15,000 (MS/MS), AGC Target: 100, HCD Collision Energy: 30%, Injection Volume: 10 µL. Data Analysis on polar metabolites were identified using MSDIAL ver.5.1.230912 with the MSMS-Publicall-neg-VS19 and MSMS-Publicexperimentspectra-neg-VS19 libraries. Data-Dependent Acquisition (DDA) was employed for untargeted identification, where precursor ions are selectively isolated and fragmented based on their intensity. This approach allows for the identification of a wide range of metabolites by generating MS/MS spectra for the most prominent ions detected in the sample. Results were manually verified to avoid mismatches. Peak area were normalized by the total protein content measured with BCA assay.

**Publicly available datasets access**

The expression of transcript visualized into the two-dimensional representation according to the integrative GBM classification model ^1^, was obtained from the Broad Institute Single Cell Portal website (https: //singlecell.broadinstitute.org/single_cell), study number SCP393 (load cell state hierarchy plot with gene expression annotation). RNA-seq gene expression and Spearman correlation were obtained from the public TCGA-GBM Dataset (<http://gliovis.bioinfo.cnio.es/>) data type HG-UG133A ^11^ exclusively considering GBM samples.

**References**

1. Neftel C, Laffy J, Filbin MG, et al. An Integrative Model of Cellular States, Plasticity, and Genetics for Glioblastoma. *Cell*. 2019;178(4):835-849.e21. doi:10.1016/j.cell.2019.06.024

2. Stanzani E, Martínez-Soler F, Mateos TM, et al. Radioresistance of mesenchymal glioblastoma initiating cells correlates with patient outcome and is associated with activation of inflammatory program. *Oncotarget*. 2017;8(43):73640-73653. doi:10.18632/oncotarget.18363

3. Bhat KPL, Balasubramaniyan V, Vaillant B, et al. Mesenchymal Differentiation Mediated by NF-κB Promotes Radiation Resistance in Glioblastoma. *Cancer Cell*. 2013;24(3):331-346. doi:10.1016/j.ccr.2013.08.001

4. Marconi S, Santamaria S, Bartolucci M, et al. Trastuzumab modulates the protein cargo of extracellular vesicles released by erbb2+ breast cancer cells. *Membranes (Basel)*. 2021;11(3):1-12. doi:10.3390/membranes11030199

5. Santamaria S, Gagliani MC, Bellese G, et al. Imaging of Endocytic Trafficking and Extracellular Vesicles Released Under Neratinib Treatment in ERBB2+ Breast Cancer Cells. *J Histochem Cytochem*. 2021;69(7):461-473. doi:10.1369/00221554211026297

6. Rai M, Curley M, Coleman Z, et al. Analysis of proteostasis during aging with western blot of detergent-soluble and insoluble protein fractions. *STAR Protoc*. 2021;2(3):100628. doi:10.1016/j.xpro.2021.100628

7. Velasco S, Kedaigle AJ, Simmons SK, et al. Individual brain organoids reproducibly form cell diversity of the human cerebral cortex. *Nature*. 2019;570(7762):523-527. doi:10.1038/s41586-019-1289-x

8. Takashimizu Y, Iiyoshi M. New parameter of roundness R: circularity corrected by aspect ratio. *Prog Earth Planet Sci*. 2016;3(1):1-16. doi:10.1186/s40645-015-0078-x

9. Siller-Jackson AJ, Burra S, Gu S, et al. Adaptation of connexin 43-hemichannel prostaglandin release to mechanical loading. *J Biol Chem*. 2008;283(39):26374-26382. doi:10.1074/jbc.M803136200

10. Lauranzano E, Pozzi S, Pasetto L, et al. Peptidylprolyl isomerase A governs TARDBP function and assembly in heterogeneous nuclear ribonucleoprotein complexes. *Brain*. 2015;138(4):974-991. doi:10.1093/brain/awv005

11. McLendon R, Friedman A, Bigner D, et al. Comprehensive genomic characterization defines human glioblastoma genes and core pathways. *Nature*. 2008;455(7216):1061-1068. doi:10.1038/nature07385
