# Supplementary figures and images for: Extracellular vesicles released by glioblastoma cancer cells drive tumor invasiveness via Connexin-43 gap junctions
Source: Neuro Oncol. 2025 Jan 30;27(11):2843–60. doi: 10.1093/neuonc/noaf013 (PMC12908494; doi:10.1093/neuonc/noaf013)

**Supplementary Fig. 1**


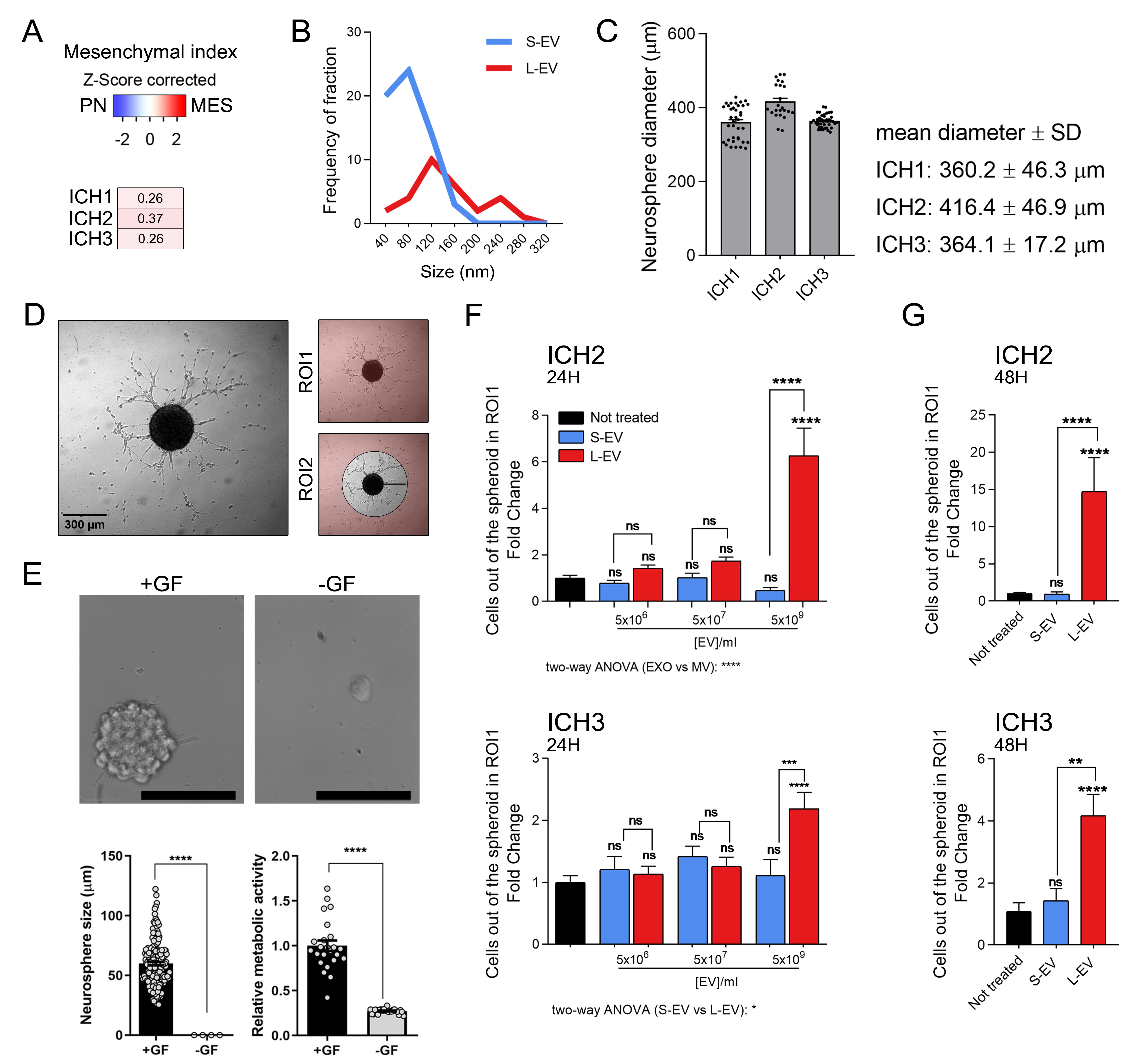


**Supplementary Fig. 2**


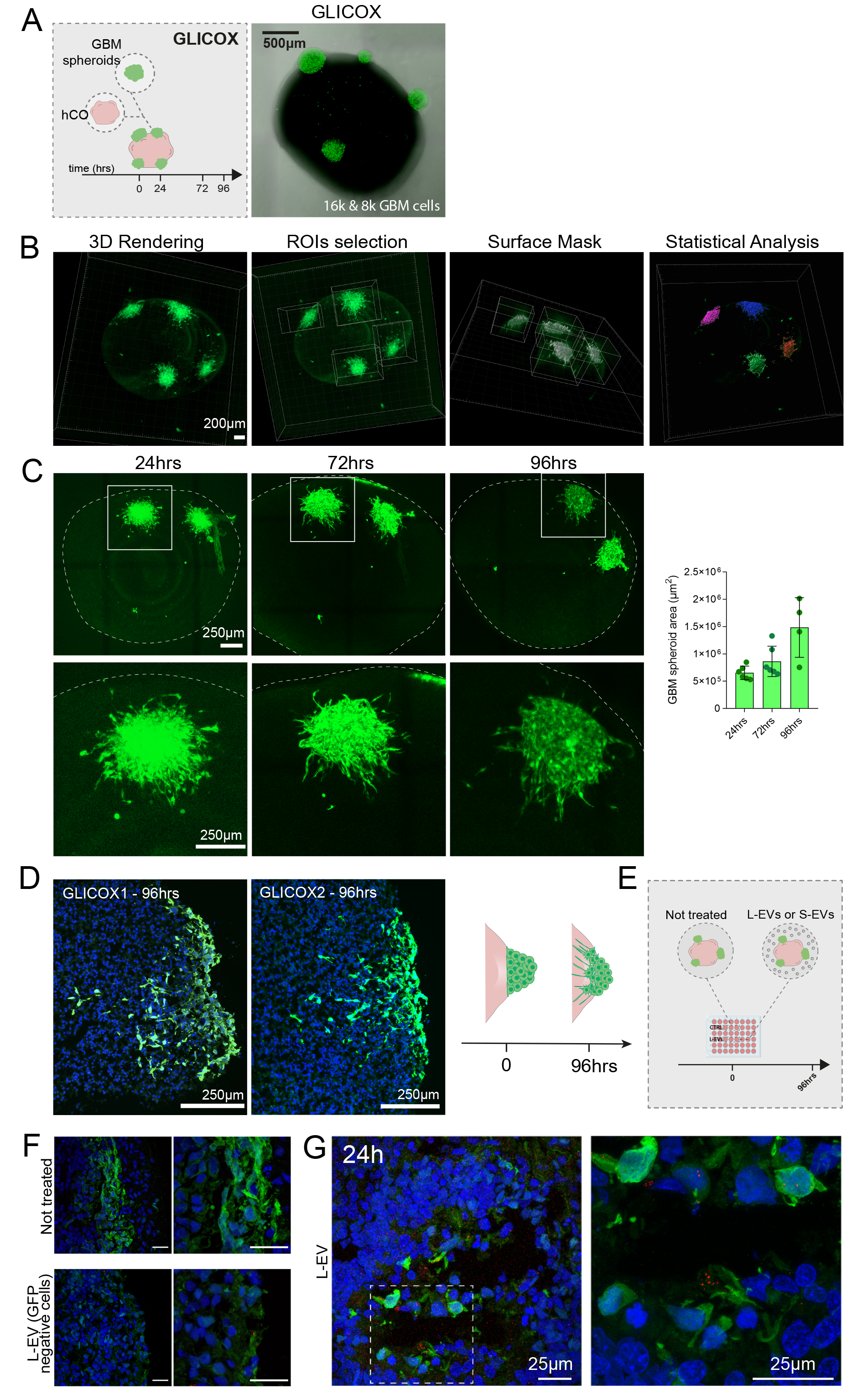


**Supplementary Fig. 3**

**
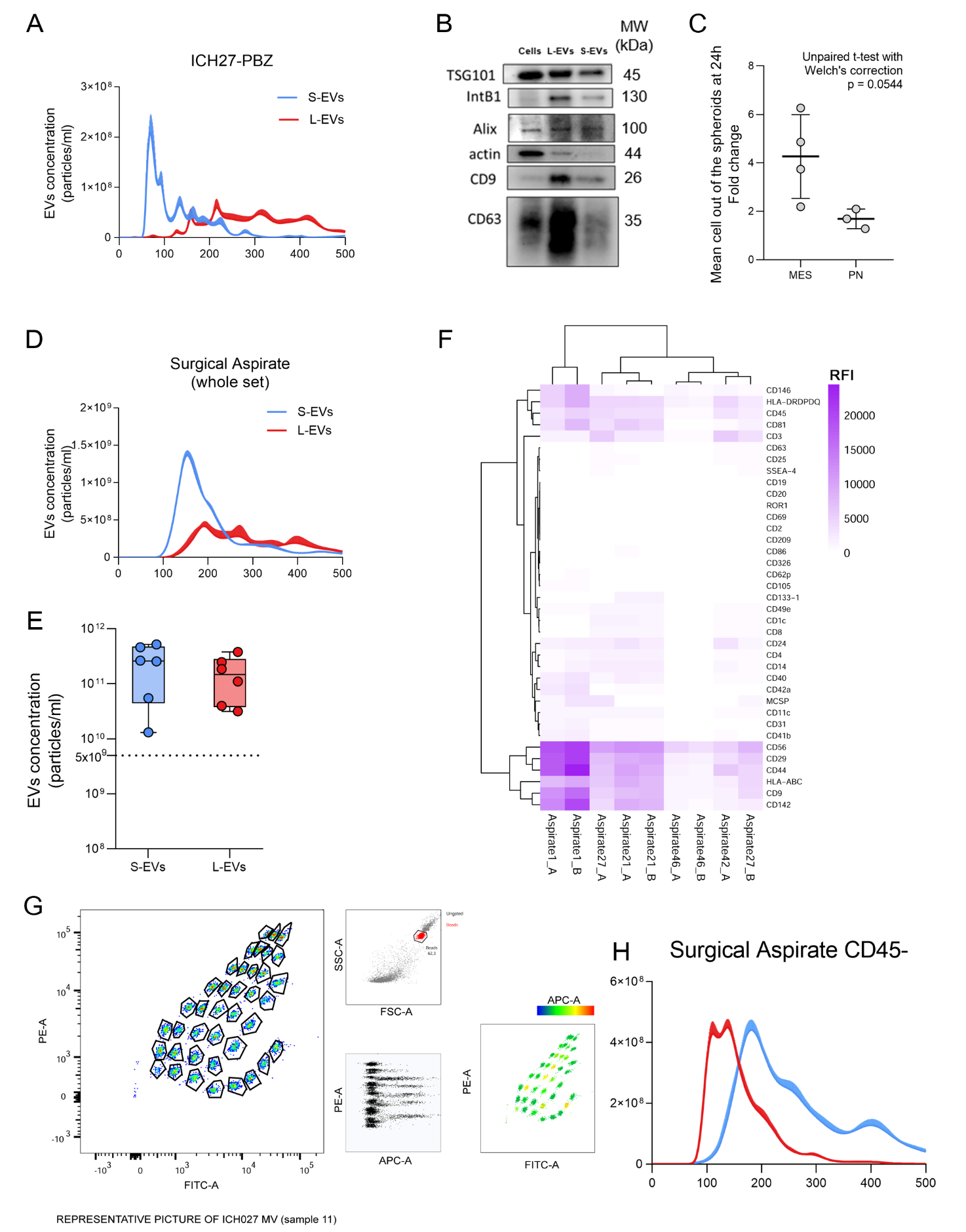
**

**Supplementary Fig. 4**

**
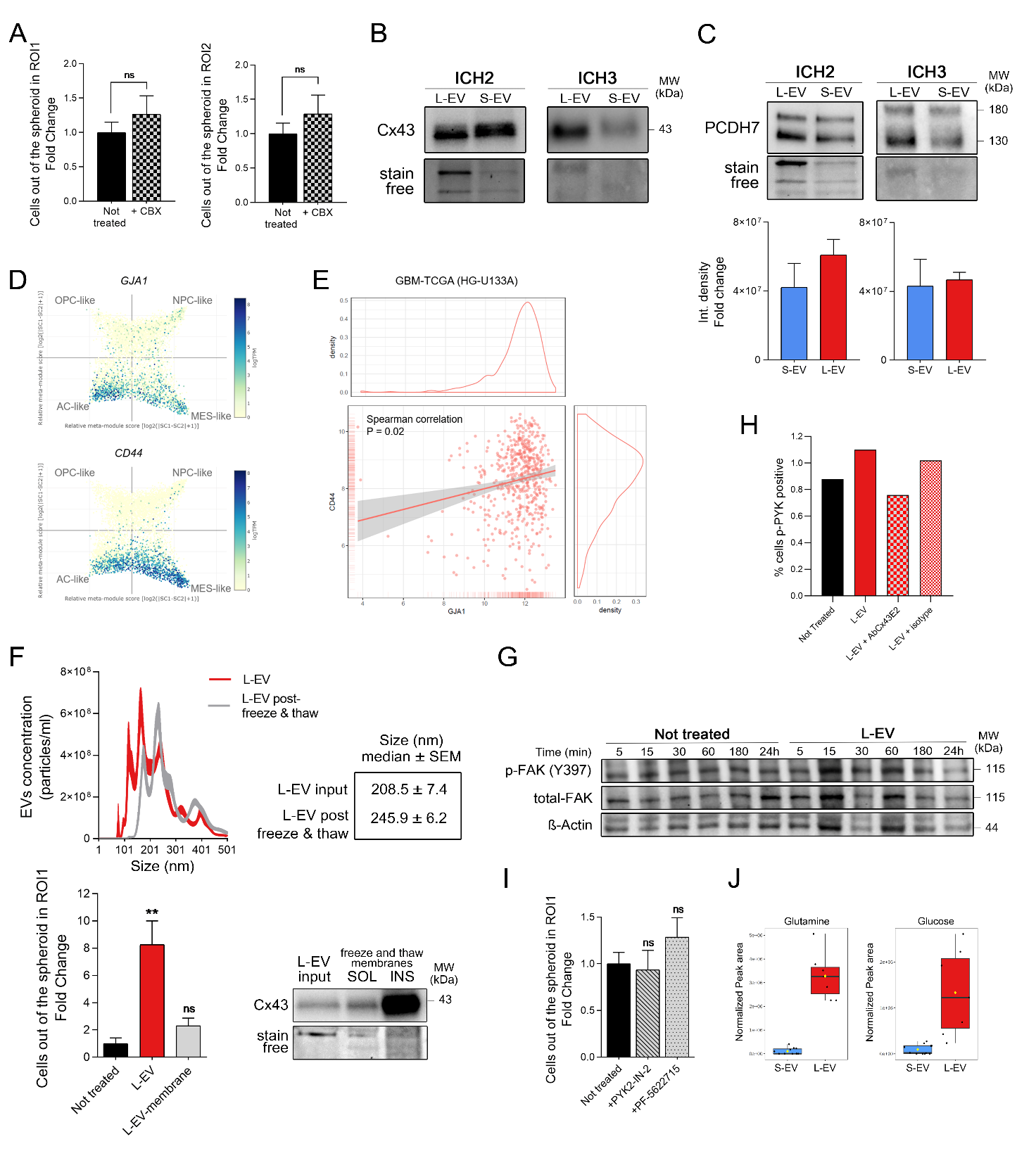
**

Supplement: noaf013_Supplementary_Figures [file noaf013_supplementary_figures.docx]
